# Metabarcoding analysis of the microbiota in flocks naturally infected by *Coxiella burnetii*: First description of the global microbiota in domestic small ruminants

**DOI:** 10.1016/j.onehlt.2025.100996

**Published:** 2025-02-19

**Authors:** R. Toledo-Perona, Á. Gómez-Martín, A. Contreras, M. Toquet, J.J. Quereda, A. Esnal, P. González-Torres, J. Gomis

**Affiliations:** a Grupo de investigación Agentes Microbiológicos asociados a la reproducción animal (ProVaginBIO), Departamento Producción y Sanidad Animal, Salud Pública Veterinaria y Ciencia y Tecnología de los Alimentos, Facultad de Veterinaria, Universidad Cardenal Herrera-CEU, CEU Universities, 46115 Valencia, Spain; b Department of Animal Health, Faculty of Veterinary Sciences, University of Murcia, 301000 Murcia, Spain; c Grupo de investigación LisBio, Departamento Producción y Sanidad Animal, Salud Pública Veterinaria y Ciencia y Tecnología de los Alimentos, Facultad de Veterinaria, Universidad Cardenal Herrera-CEU, CEU Universities, 46115 Valencia, Spain; d Analítica Veterinaria - Mungivet S.L., 48100 Mungia, Bizkaia, Spain; e Microomics Systems S.L., 08041 Barcelona, Spain

**Keywords:** Q fever, Zoonoses, Sheep, Goats, Environmental, Microbiome, Dysbiosis

## Abstract

This study investigates Q fever in sheep and goats, key reservoirs for human infection, by metabarcoding and comparing it with q-PCR and serology. Samples from 26 small ruminants (aborted and normal-delivery) and six males across three Q fever-affected herds were analyzed. In sheep herds, seropositivity was 50 and 80 % respectively, with *Coxiella (C.) burnetii* shedding detected vaginally in the second herd. In goats, 100 % seropositivity and 90 % *C. burnetii* detection were observed, with nasal and vaginal samples showing the highest detection rates. Metabarcoding revealed significant differences in alpha diversity, with greater richness in blood and evenness in milk from normal-delivery sheep and higher evenness in faeces from aborted sheep. Beta diversity showed distinct vaginal microbiota in normal-delivery females compared to aborted ones. Firmicutes was the most abundant phylum observed. Dominant genera included: *Moraxella* (nasal), *Mycoplasma* (blood), *Streptococcus* (milk), *Ureaplasma* (vaginal and preputial), *Rikenellaceae RC9 gut group* (faeces). Significant differences in bacterial composition, including infertility-linked vaginal pathogens, were found across female groups in all herds in the anatomical locations studied, revealing new species and tropisms. Moreover, taxonomic analysis identified *C. burnetii* in vaginal, milk and environmental samples. This first report of *C. burnetii* in the caprine nasal cavity suggests an underestimated tropism that may improve Q fever diagnosis. These findings underscore the need for herd-wide Q fever control measures, including males and normal-delivery females. Our findings contribute to new insights into the pathogen's impact on small ruminant microbiota and a novel approach to studying infectious diseases in this sector.

## Highlights

• The entire herd must be included in Q fever control and prevention measures.
• An underestimated *C. burnetii* tropism of caprine nasal cavity has been suggested.
• Abortion showed a negative impact on the studied microbiota.
• Abortion causes significant differences in bacterial genera and species.
• The axis environment-host microbiota should be taken into consideration.

## 1 Introduction

Q fever is a highly contagious zoonotic disease with worldwide distribution [1]. *Coxiella burnetii* (*Cb*), the causal agent, is a Gram-negative obligate intracellular bacterium. The endospore-like structure-resistant form called small cell variant (SCV) provides them with the ability to survive extreme environmental conditions [2], and persist for long periods in soil, manure and dust. Many mammals, birds, reptiles and arthropods are reservoirs of Q fever [3]. However, goats and sheep are considered the main source of *Cb* infection for humans [4]. Globally, an average prevalence of 2–19 % in sheep, and 2.5–66 % in goats has been estimated [5,6]. In Spain, the lack of standardized studies hinders accurate evaluation of Q fever prevalence in small ruminants, though some report higher incidence in the North compared to other regions [7]. The relevance on public health and the economic impact on livestock [8], especially for the ovine and caprine industry [3,9,10], places special importance on this disease. The main symptoms in small ruminants include an increase in abortions or stillbirths during late pregnancy and the delivery of weak neonates [3,9,11]. Sheep and goats infected by *Cb* can be asymptomatic, therefore Q fever is sometimes an underdiagnosed disease in flocks, thus increasing the risk of transmission of the bacteria [6].

Regarding transmission routes, milk, vagina and faeces in goats, and faeces in sheep are the main routes of excretion [11,12]. Bacterial spread in the environment primarily occurs after delivery or abortion [13,14] and can persist for several weeks in symptomatic or asymptomatic females [4,15]. Inhalation of dust and aerosols is the main route of transmission to humans and the most natural via of infection in animals [16]. The respiratory tropism of *Cb* in humans is well-known, as it has been involved in pneumonia cases [5,16]. Nevertheless, a lack of information in terms of respiratory tropism in small ruminants exists. To date, some authors have reported the presence of *Cb* in the ovine respiratory tract, but its detection has been related to environmental contamination [17]. The potential underestimation of respiratory tropism underscores the need for further studies on disease control and prevention in small ruminants.

Additionally, sexual transmission of Q fever cannot be excluded, as *Cb* has been detected in semen of ram, bull and mouse [[18], [19], [20]]. However, information about the male's role in the epidemiology of this disease in small ruminants is limited. The identification of the bacteria in semen and its great environmental persistence in the bedding [21] led us to hypothesize that *Cb* may be part of the foreskin microbiota in males, highlighting the need for targeted control and prevention strategies on them.

Vaccination, antibiotic therapy and biosecurity are common measures against Q fever in domestic ruminant herds. Epidemiological differences between ovine and caprine species, asymptomatic carriers, the limited information on respiratory tropism and the role of rams and bucks raise questions such as the best samples for diagnosis and which groups to vaccinate, treat, and isolate (males/females; symptomatic/asymptomatic). In Q fever surveillance, the most employed tools for detecting *Cb* in a herd are serology and molecular techniques such as q-PCR [22]. Nevertheless, innovative techniques such as metabarcoding could provide new approaches to studying Q fever epidemiology in small ruminants, as they have not been previously used. These ‘omics’ studies have been used in small ruminants for the respiratory, digestive and dairy microbiota description [[23], [24], [25], [26]], as well as for the reproductive microbiota in females and males [[27], [28], [29], [30]]. Moreover, metagenomics can give us information about bacterial interactions, the presence of bacterial populations associated with healthy status and fertility [31] or even the interaction between vaginal and preputial microbiota in small ruminants [27,32]. Indeed, studying the microbiota of different anatomical locations, such as the respiratory and digestive system [33], clarifies their connections and enhances our understanding of diseases in domestic animals.

To the authors' knowledge, no global study of small ruminant and environmental microbiota, nor the impact of *Cb* on mammalian microbiota, even in humans, has been conducted. The present study is based on the following hypotheses: 1) Control and prevention measures should be applied to the entire herd, regardless of clinical condition or sex; 2) Respiratory tropism in small ruminants may be underestimated; 3) *Cb* may cause previously unconsidered changes in the ovine and caprine microbiota. Therefore, the objective of the present study was to evaluate the presence of *Cb* in Spanish ovine and caprine herds situated in the eastern of Iberian Peninsula where this pathogen circulates as well as the microbiota associated with it. For this purpose, samples from the vaginal, preputial and nasopharyngeal samples, as well as raw milk and faeces were obtained from unvaccinated herds for diagnosis by q-PCR and metabarcoding analysis. This last technique was also used on blood samples in combination with a serological diagnosis by ELISA. Environmental samples were also obtained and analyzed with both molecular techniques.

## 2 Material and methods

### 2.1 Study population

A total of 32 small ruminants (26 females and six males) from one caprine and two ovine herds were included in the study (Table 1). All of them were situated in the east of the Iberian Peninsula. The flocks were selected based on high and diverse abortion rates associated with Q fever, with *Cb*-seropositive animals and pathogen detection. Specifically, *Cb* PCR-positive samples from placenta tissue and vaginal swabs were obtained from aborted females one week before starting the study. Herd A (goats) presented an abortion outbreak with birth of weak kids and the death of adult goats. Anecdotally, some vaginal samples were also *Chlamydia (C.) abortus* positive. This herd was a newly created flock with the introduction of nulliparous pregnant goats (Table 1). Herd B (dairy sheep) showed a less severe clinical outbreak of abortions where samples were positive for *Toxoplasma gondii* too. In that herd, *Listeria (L.) monocytogenes* was seasonally detected in faecal samples two years ago [34], but no abnormal abortion episodes were observed during that study period. Finally, in herd C (meat sheep), abortion rates of 10 % were observed and no other abortive etiological agents were identified. No antibiotic treatment was administered to the study animals before sample collection. Finally, all herds had a *C. abortus* vaccination program, as well as one against *Toxoplasma gondii* in the case of herd B (dairy sheep). Despite this, no Q fever vaccination program was implemented in any of the studied herds.

**Table 1:** Characteristics of the three sampled herds.

	Herd A^1^	Herd B^2^	Herd C^3^
Sampling season	Spring	Winter	Winter
Species	Goat	Sheep	Sheep
Breed	Murciano-Granadina	Lacaune	Mixed-meat breed
Production system	Intensive	Intensive	Semi-extensive
Breed aptitude	Dairy	Dairy	Meat
Total census	2000	2000	8000
(%) Abortion rate^4^	70	40	10
Weak kids	Yes	No	No
Recent entry of new animals	Yes^5^	No	No
Presence of domestic animals	No	Yes	Yes
Type of troughs	Metal hay rack	Ground	Concrete

1 Goats.

2 Dairy sheep.

3 Meat sheep.

4 During last lambing/kidding.

5 New herd created through the purchase of nulliparous goats.

### 2.2 Ethics approval

The study protocol was reviewed and approved by the Animal Welfare & Ethics Committee of CEU Cardenal Herrera University (Alfara del Patriarca, Spain) by the Spanish Regional Government Generalitat Valenciana (Alfara del Patriarca, Spain; 2024-VSC-PEA-0120).

### 2.3 Sampling approach

The study population (22 sheep and 10 goats) from each herd was divided into three experimental groups: group 1 (G1), aborted females; group 2 (G2), normal-delivery females; group 3 (G3), males (Table 2). Females were sampled during the first week after abortion/delivery. For sample collection, at least two researchers assisted with animal immobilization and sampling. Personnel wore personal protective equipment (sterile gloves, FFP3 masks, disposable waterproof coveralls, and boot covers), changing gloves between samples to prevent cross-contamination. Blood, faeces, individual raw milk, and nasal, vaginal or preputial swabs were obtained per animal. Blood was obtained by venipuncture of the jugular (Vacutainer® SST, 5 mL serum separation tube). After that, it was centrifugated at 10,000*g* for 15 min and the serum fraction was frozen until the serological study was performed [17]. In addition, vaginal, preputial and nasal swabs, faeces, blood, individual milk, and environmental samples were obtained to carry out metagenomic analysis. All these same samples, except blood, were also collected to detect *Cb* DNA by q-PCR. The external skin was cleaned and disinfected with chlorhexidine 2 % and vaginal, preputial and nasopharyngeal samples were hygienically taken with a sterile DNA-free cotton swab (Deltalab®-ref. 300,263) for metagenomic analysis and with AMIES PS + VISCOSA swabs (Deltalab®-ref. 300,287) for q-PCR diagnosis. Swab samples were obtained by gently swabbing the internal mucosa of the deep vagina, preputial sac, and nasopharynx following the methodology of previous studies [27,35,36]. Then it was extracted carefully, avoiding contact with the external skin, and it was introduced in the transport swab tubes. To perform milk sampling, the right teat was always used. A California Mastitis Test (CMT) (KerbaTest, KERBL®) was performed in the sampled udder of all the females before milk sampling. Three scores were established for the CMT interpretation: negative (−), low positive (+), medium positive (++), and high positive (+++) following the methodology of [37]. Before milk sampling, the teat was previously disinfected with 70 % alcohol and dried with a sterile gauze, followed by the discard of the first milk. Faecal samples were collected directly from the rectum of all animals by hand covered with a sterile glove. Environmental samples and faeces from other domestic animals (chickens) were additionally included for metabarcoding and q-PCR analysis. One bedding sample from different delivery zone locations (organic matter from the surface to about 10 cm deep) were also taken from each herd [21]. Moreover, in the same locations, one trough swab from each farm was obtained. In the herds B and C, domestic chicken faecal swabs were sampled due to the proximity of them to the sheep and the possible transmission by the farm workers. Sterile cryovials (Deltalab®-ref. 409,106.1) were used for faeces, milk and environmental samples as described in previous studies [38,39]. Samples were kept at -20 °C for q-PCR and serologic analysis, and at -80 °C for metagenomic samples.

**Table 2:** Individual serological and molecular results in herd A, B and C.

	Herd A^1^										Herd B^2^												Herd C^3^									
	G1				G2				G3		G1						G2				G3		G1				G2				G3	
Animal	**1**	**2**	**3**	**4**	**5**	**6**	**7**	**8**	**9**	**10**	**1**	**2**	**3**	**4**	**5**	**6**	**7**	**8**	**9**	**10**	**11**	**12**	**1**	**2**	**3**	**4**	**5**	**6**	**7**	**8**	**9**	**10**
N° parities	P	P	P	P	P	P	P	P	/	/	P	P	P	P	P	P	M	P	P	M	/	/	P	P	P	P	M	M	M	M	/	/
Serology	+	+	+	+	+	+	+	+	+	+	+	–	+	+	+	+	+	+	+	+	–	+	+	+	–	–	–	+	–	+	+	–
Nasal	+	+	+	–	+	+	+	+	+	–	–	–	–	–	–	–	–	–	–	–	–	–	–	–	–	–	–	–	–	–	–	–
Vaginal/ Preputial	+	+	+	+	+	+	+	+	–	–	–	+	–	–	–	–	–	+	+	–	–	–	–	–	–	–	–	–	–	–	–	–
Faeces	+	–	–	–	+	+	+	+	–	–	–	–	–	–	–	–	–	–	–	–	–	–	–	–	–	–	–	–	–	–	–	–
Milk	–	–	–	–	+	–	–	–	/	/	–	–	–	–	–	–	–	–	–	–	/	/	–	–	–	–	–	–	–	–	/	/

G1, aborted females; G2, normal-delivery females; G3, males; P, primiparous female; M, multiparous female; −, negative result; +, positive result; /, not relevant.

1 Goats.

2 Dairy sheep.

3 Meat sheep.

A total of 32 blood samples were used for serologic analysis. For q-PCR a total of 130 samples were obtained (32 faeces, 32 nasopharyngeal swabs, 26 vaginal swabs, 26 raw milk samples, six preputial swabs, three trough swabs, three bedding samples and two domestic chicken faecal swabs). For metagenomics analysis, 162 samples were analyzed, including the same type of samples used for q-PCR diagnosis, as well as to 32 blood samples.

### 2.4 Molecular analyses: DNA extraction and real-time PCR

DNA was extracted using a commercial kit (MagMAX CORE Nucleid Acid Purification Kit, Applied Biosystems, Thermo Fisher Scientific®, Ref. A32702) from swabs, faecal, milk, and environmental samples, following the manufacturer's instructions for low-input workflow. Swab samples were suspended in 1 mL of PBS and, after agitation for 3 min, the supernatant (200 μL) was collected. Milk samples were processed directly (200 μL). Faecal and environmental samples (0.3–0.4 g) were suspended in 1 mL of PBS, agitated for 3 min, and centrifuged at 100*g* for 1 min, with the supernatant (200 μL) subsequently collected. Once the samples were prepared, they were processed according to the manufacturer's instructions for the extraction kit, which is based on magnetic bead separation, using the Automated Nucleic Acid Purification System Zixpress 32 (Zinexts Life Science Corporation). The presence of *Cb* DNA was investigated by a real-time PCR procedure targeting the transposon-like repetitive region *IS1111* of *Cb* genom. q-PCR were performed by a commercial kit (*Coxiella burnetii* monodose DTEC-qPCR with internal control, GPS Genetic Analysis Strategies®) [[40], [41], [42], [43]]. Samples with *Ct* < 37 were considered positive, and inconclusive result were samples with *Ct* > 37.

### 2.5 Serological analyses: Enzyme-linked immunosorbent assay

To evaluate seropositive animals against *Cb*, serum samples were tested for Q fever antibodies using an ELISA test (*Coxiella burnetii* Monoscreen Ab-ELISA. BIO-X DIAGNOSTICS® K 298/2). The entire 96-well microplates were sensitized with antigenic extracts of *Cb* in phase I + II. After 60 min of incubation and a washing step, the protein G conjugated to peroxidase was added. After 60 min of incubation and a washing step, the chromogen tetramethylbenzidine (TMB) was added. The blood sera samples and kit controls (the positive, negative controls and the tracer) were diluted 100-fold in the dilution solution and homogenized (10 μL of sample + 990 μL of dilution solution). All the reagents were at 21 ± 3 °C before use. For the serum protocol (1/100 dilution), 100 μL were distributed per well of diluted serum samples and kit controls. After that, the plate was covered and incubated at 21 ± 3 °C during 60 ± 5 min. After the incubation, the content of the microplate was removed, and the microplate was washed three times with 300 μL of washing solution per well and 100 μL of diluted conjugate were added per well. Again, the plate was covered with a lid and incubated at 21 ± 3 °C during 60 ± 5 min. After 5 min, the content of the microplate was removed, and the microplate was washed three times with 300 μL of washing solution per well again. The TMB solution was distributed in 100 μL per well and incubated at 21 ± 3 °C during 10 ± 1 min away from the light, without covering. After the minute, 50 μL of the stopping solution were distributed per well. Positive sample color changed from blue to yellow. Finally, the optical density (OD) was recorded using a plate spectrophotometer with a 450 nm filter within 5 min after adding the stopping solution. For the validation of the results, the test can only be validated in two situations: 1) the difference between positive and negative serum OD readings is greater than 1000 (OD positive serum - OD negative serum >1000); 2) the negative serum optical density reading is less than 0.400 (OD negative serum <0.400). For the results interpretation, the coefficient (S/P %) was calculated for the result's obtention for each sample using the formula:

$$S/P\%=\frac{\mathrm{OD}\;\text{sample}‐\mathrm{OD}\;\text{negative serum}}{\mathrm{OD}\;\text{positive serum}‐\mathrm{OD}\;\text{negative serum}}*100$$

A ratio ELISA-Ac IgG was calculated for the result's obtention. Negative samples were considered when S/P % < 40 %, doubtful samples when the results were 40 % ≤ S/P % ≤ 60 %, and finally, positive results were S/P % > 60 %.

### 2.6 Evaluation and accordance of diagnosis methods

To assess the different diagnostic methods or samples employed, we conducted validation and concordance tests for the different methods (q-PCR or serology) and samples used (nasopharyngeal, vaginal/preputial, faeces or milk). Both processes were carried out using the WinEpi program [44], with a 95 % confidence level. Assuming the absence of false positives in the q-PCR against *Cb* in the analyzed samples, we created a variable that compiles the positive results from any of them to be used as a Gold Standard for validating the different diagnostic methods employed and to calculate the validity parameters for the diagnostic techniques (sensitivity and specificity), as well as positive and negative predictive values, Youden's J, and fiability. The concordance level between methods was performed by the Kappa test. Due to the limited number of preputial samples, were considered ‘genital samples’ the total of vaginal and preputial samples for the validation and concordance test. It was studied by a Chi-square test with a 95 % confidence level the existence of significant differences between G1 (aborted females) and G2 (normal-delivery females) in the three herds.

### 2.7 Metabarcoding analyses

#### 2.7.1 Library preparation and sequencing

A marker-based approach using the 16S ribosomal RNA subunit gene (16S rRNA) was used to study bacterial diversity of 162 samples. This approach enabled a description and quantification of the microbial alpha and beta diversity and the study of taxonomic profiles from the phylum to species level.

DNA extraction of swabs samples, milk and blood were performed following a methodology previously described [27]. Faecal sample DNA extraction was performed using a commercial kit (MagMAX CORE Nucleid Acid Purification Kit, Applied Biosystems, Thermo Fisher Scientific®, Ref. A32702) with the extraction equipment (ZIXpress 32. Zinexts Life Science Corporation). The quality ratios (260/230) and (260/280) as well as the concentration levels (ng/ul) of DNA obtained were within the usual ranges for this type of samples, therefore the extraction yields were satisfactory. A mock community DNA was included as positive control for library preparation (Zymobiomics Microbial Community DNA, Catalog Nos. D6305, ZymoResearch, Irvine, CA, United States). Samples were amplified using primers specific to the V3–V4 regions of the 16S rRNA DNA (V3–V4-Forward5′TCGTCGGCAGCGTCAGATGTGTATAAGAGACAGCCTACGGGNGGCWGCAG-3′, V3–V4 Reverse 5′GTCTCGTGGGCTCGGAGATGTGTATAAGAGACAGGACTACHVGGGTATCTAATC-3′). Amplification was performed after 25 PCR cycles for faecal, swabs samples, milk and blood samples following a methodology previously described [27]. Amplification of the mock community standard was expected, 450 bp-size amplicons were obtained. After the second PCR, 25 μl of the final product was used for purification and normalization with SequalPrep normalization kit (Invitrogen), according to manufacturer's protocol. Libraries were eluted in 20 μl volume and pooled for sequencing. Sequencing was performed using Illumina MiSeq with 2 × 300 bp reads using v3 chemistry with a loading concentration of 10 pM. In all cases, 15 % of PhIX control libraries were used to increase the diversity of the sequenced sample.

Negative controls included sample collection buffer, DNA extraction, and PCR amplification steps, PCR products after both PCR steps were visualized using an electrophoresis gel (1.5 % agarose) with SYBR Safe (Applied Biosystems, ThermoFisher Scientific, Waltham, MA, United States). No visible bands were observed. A positive Mock Community control was also included to ensure quality control.

#### 2.7.2 Bioinformatics processing and analysis

Raw demultiplexed forward and reverse reads were processed using QIIME2 version 2019.4 with default parameters unless stated [45]. DADA2 was used for reading trimming, quality filtering, denoising and pair-end merging, and phylotype calling [46]. The achieved sequencing depth and subsampling size were enough to observe the complete diversity present in the microbial communities. Q20 was used as quality threshold to define read sizes for trimming before merging (parameters: –p-trunc-len-f and –p-trunc-len-r). Reads were truncated at the position when the 75th percentile Phred score felt below Q20: 300 bp for forward reads and 242 bp for reverse reads. After quality filtering steps, the average sample size was 33,144.8 reads (min: 13,680 reads, max: 58,336 reads). Three were performed three types of comparations: Comparison 1, comparisons between G1 (aborted females) and G2 (normal-delivery females) from the same herd; Comparison 2: comparisons between the three G1 (aborted females) and the three G2 (normal-delivery females) groups from the three herds (A, B, C); Comparison 3: comparisons between females depending on the animal species (sheep vs. goat).

Phylotype data was used to calculate the following alpha diversity metrics: community richness (observed Amplicon Sequence Variants, ASVs) and evenness (Pielou's evenness index). Alpha diversity comparisons were performed using a Generalized Linear Model, the R package MASS v.7.3-54 [47] was used for richness and the R package glmmTMB v.1.1.8 [48] was used for evenness. If a Generalized Linear Mixed Model was calculated, the R package NBZIMM v.1.0 [49] was used for richness and the R package betareg v.3.1-4 [50] for evenness. Significant threshold was set at 0.05. ASVs were aligned using the qiime alignment mafft meth [51]. The alignment was used to create a tree and to calculate phylogenetic relations between ASVs using qiime2 phylogeny fasttree method [52]. ASV tables were subsampled without replacement in order to even sample sizes for diversity analysis using qiime diversity core-metrics-phylogenetic pipeline. The smallest sample size was chosen for subsampling [53]. ASVs and phylogenetic data were used to calculate the following beta diversity metrics: unweighted UniFrac, weighted UniFrac, Jaccard, Bray-Curtis. Beta diversity distance matrices were used to calculate principal coordinates analysis (PCoA) and to make ordination plots using R software package version 4.2.0. The significance of groups was tested using Permanova and ANOSIM tests. Permdisp test was used to identify location vs. dispersion effects [54]. A significant threshold was set at 0.05.

Taxonomic assignment of ASVs was performed using a Bayesian Classifier [55] trained with Silva database version 138 (99 % ASVs full-length sequences) using the qiime feature-classifier classify-sklearn method [56]. Differential abundance of taxa was tested using Negative Binomial Generalized Linear Models. Either a Generalized Linear Model using the R package MASS v.7.3-54 [47] or a Generalized Linear Mixed Model using the R package NBZIMM v.1.0 [49] were calculated. Significant threshold was set at 0.05. BiodiversityR version 2.14-1, PMCMRplus version 1.9.4, RVAideMemoire version 0.9-8 and vegan version 2.5-6 packages were used for the different statistical analysis carried out. The taxonomic profile of the mock community control matched the expected bacterial profile.

## 3 Results

### 3.1 Serological and q-PCR results

Serological and q-PCR results are shown in Table 2. No differences in q-PCR results (*P* > 0.05) were observed between both groups of females. Differences in serological results and between species could not be analyzed due to the insufficient number of observations (< 5), which does not allow to perform a valid analysis. Herd A (goats) showed the highest level of seropositivity and *Cb* detection in comparison to herds B and C (caprine total seropositivity was 100 %, 10/10; ovine total seropositivity was 68.18 %, 15/22). According to the sex, males showed a seropositivity of 67 % (4/6) and 80.7 % (21/26) in females. A Chi-square test was conducted to assess seropositivity differences between female's groups from each herd and it revealed no significant differences (*P* > 0.05) for the serological results. Regarding molecular diagnosis by q-PCR, *Cb* was detected in 90 % (9/10) of goats in herd A. On the contrary, only 13.6 % (3/22) of sheep (herds B and C) were positive by q-PCR. Depending on the type of analyzed sample, 80 % of nasopharyngeal and vaginal samples were positive in goats (herd A). Faecal (50 %, 5/10) and milk (25 %; 3/12) positive samples were observed only in the goat herd. In sheep, only vaginal samples were positive (25 %; 3/12) from herd B (dairy sheep). Herd C (meat sheep) did not have any q-PCR positive samples. Although vaginal samples were the most frequently detected positive sample, no significant differences were observed in positivity based on sample type (*P* > 0.05). Comparing experimental groups in the caprine farm, normal-delivery females (G2) from herd A showed a higher number of q-PCR positive samples (13/16) compared to aborted females (8/16) and males (1/6), the last corresponding to a nasopharyngeal sample from a seropositive buck. In herd B (dairy sheep), G2 (normal-delivery females) also presented a higher number of q-PCR positive samples (2/16) than in aborted females (1/16) and males (0/6). Regarding environmental samples, bedding samples in herds A (goats) and B, and the troughs swab in herd A were q-PCR positive. None of the environmental samples from herd C (meat sheep) were q-PCR positive. The CMT results showed that a total of 12 females (46.14 %) had a negative result (-), six females (23.10 %) presented low subclinical mastitis (+), four females (15.38 %) presented a moderate status (++), and finally, four sheep (15.38 %) showed high subclinical mastitis (+++). Herd B (dairy sheep) exhibited the highest percentage of animals with a positive CMT result (27 %) compared to the other two herds (3.85 % herd A; 23.10 % herd C). The *Cb*-positive goat milk sample was negative to CMT.

A validation study was performed for the serologic and molecular (q-PCR) techniques depending on the type of samples (Table 3). The serology and the genital swabs were the most sensitive methods for *Cb* detection (91.70 %), followed by nasal swabs (66.7 %). Regarding specificity, it was higher for samples analyzed by molecular analysis compared to serologic methods. Finally, the agreements among different sample types in the study are shown in Table 4. Based on the criteria established [57], notable agreement was observed between nasal swabs with vaginal swabs (0.669) and with faeces (0.712). For the remaining samples, the agreement was poor.

**Table 3:** Validation study for the study population (95 % confidence interval) expressed in percentage (%).

	Sensitivity	Specificity	PPV	NPV	AP^1^	Youden's J	Fiability
Serology (ELISA)	91.7	30	44	85.7	78.1	21.7	53.1
Nasal swab	66.7	100	100	83.3	25	66.7	87.5
Genital swabs^2^	91.7	100	100	95.2	34.4	91.7	96.9
Faeces	41.7	100	100	74.1	15.6	41.4	78.1
Milk	8.3	100	100	64.5	3.1	8.3	65.6

Nasal, genital, faeces and milk were analyzed by q-PCR; ^1^The prevalence reference (gold standard) was 37.50 %; ^2^Including vaginal and preputial samples; PPV, Positive Predictive Value; NPV, Negative Predictive Value; AP, Apparent Prevalence.

**Table 4:** Concordance results for serology and q-PCR results depending on the type of sample.

Concordance studied	Kappa coefficient
Serology-q-PCR	0.178
Nasal swab-serology	0.171
Nasal swab-genital	0.545
Nasal swab-faeces	0.712
Nasal swab-milk	0.200
Serology-genital	0.150
Serology-faeces	0.099
Serology-milk	0.019
Genital^1^-faeces	0.522
Vaginal-milk	0.103
Faeces-milk	0.288

1 Including vaginal and preputial samples. 95 % confidence limit.

### 3.2 Metagenomic results

The total number of pair-end reads obtained from each herd, experimental group and species depending on the type of sample is shown in the Supplementary Table S1. The results from the alpha and beta diversity analyses from the three herds and species are described in Supplementary Table S2 and Supplementary Table S3.

#### 3.2.1 Diversity analysis: Alpha diversity

**Fig. 1:**
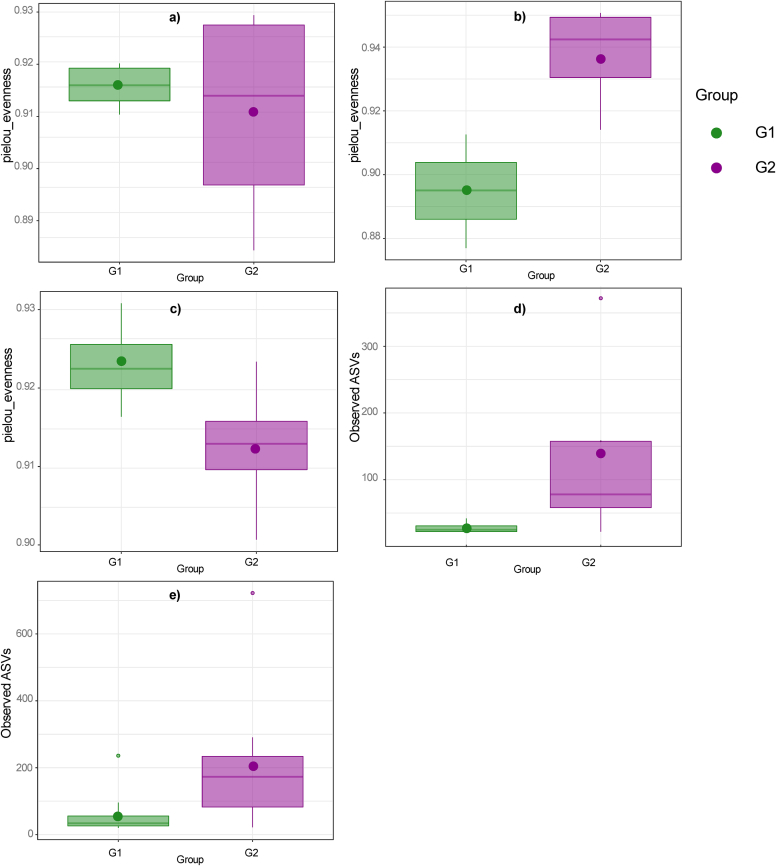
Significant differences (*P* < 0.05) in the alpha diversity (richness or evenness) between G1 (abortion) and G2 (normal-delivery) within a herd (a,b,c,d) and between G1 and G2 of the three herds (e) depending on the sample. a) Faecal samples (herd B); b) Milk samples (herd B); c) Faecal samples (herd C); d) Blood samples (herd C); e) Vaginal samples (G1 vs. G2).

**Fig. 2:**
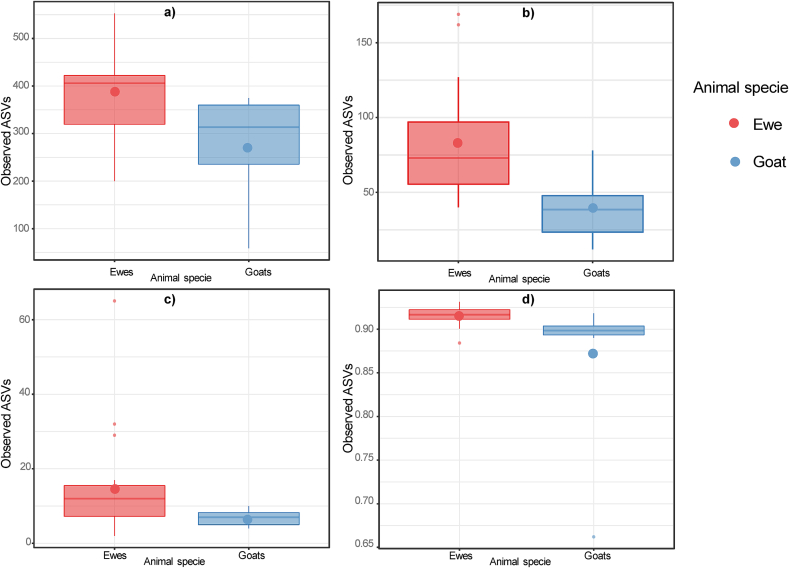
Significant differences (*P* < 0.05) in the alpha diversity (richness) analysis in the comparison between species (goat vs. sheep) depending on the sample. a) Faecal samples; b) Nasal samples; c) Blood samples; d) Faecal samples.

Rarefaction plots showed that the achieved sequencing depth and subsampling size were enough to observe the complete diversity of microbial communities in the samples since a plateau was reached. Alpha diversity showed significant differences between female groups in sheep herds (B and C) for the community richness and Pielou's evenness indices (Supplementary Table S2). No significant differences were observed in the alpha diversity of herd A (goats). In herd B (dairy sheep), Pielou's evenness index was significantly higher in in faecal samples from aborted females (*P* < 0.05) and in milk samples for normal-delivery females (*P* < 0.001) (Fig. 1a and b, respectively). In herd C (meat sheep), community evenness was significantly higher in faecal samples (*P* < 0.05) in aborted females, and richness index was significantly higher in normal-delivery females blood samples (*P* < 0.01) (Fig. 1c and d, respectively). Alpha diversity comparisons between the three aborted and normal-delivery groups from each herd (comparison 2) highlight higher community richness in vaginal samples from normal-delivery females than in aborted ones (*P* < 0.0001) (Fig. 1e). Alpha diversity differences between animal species indicated higher richness in faecal, nasal and blood samples from sheep (Fig. 2).

#### 3.2.2 Diversity analysis: Beta diversity

**Fig. 3:**
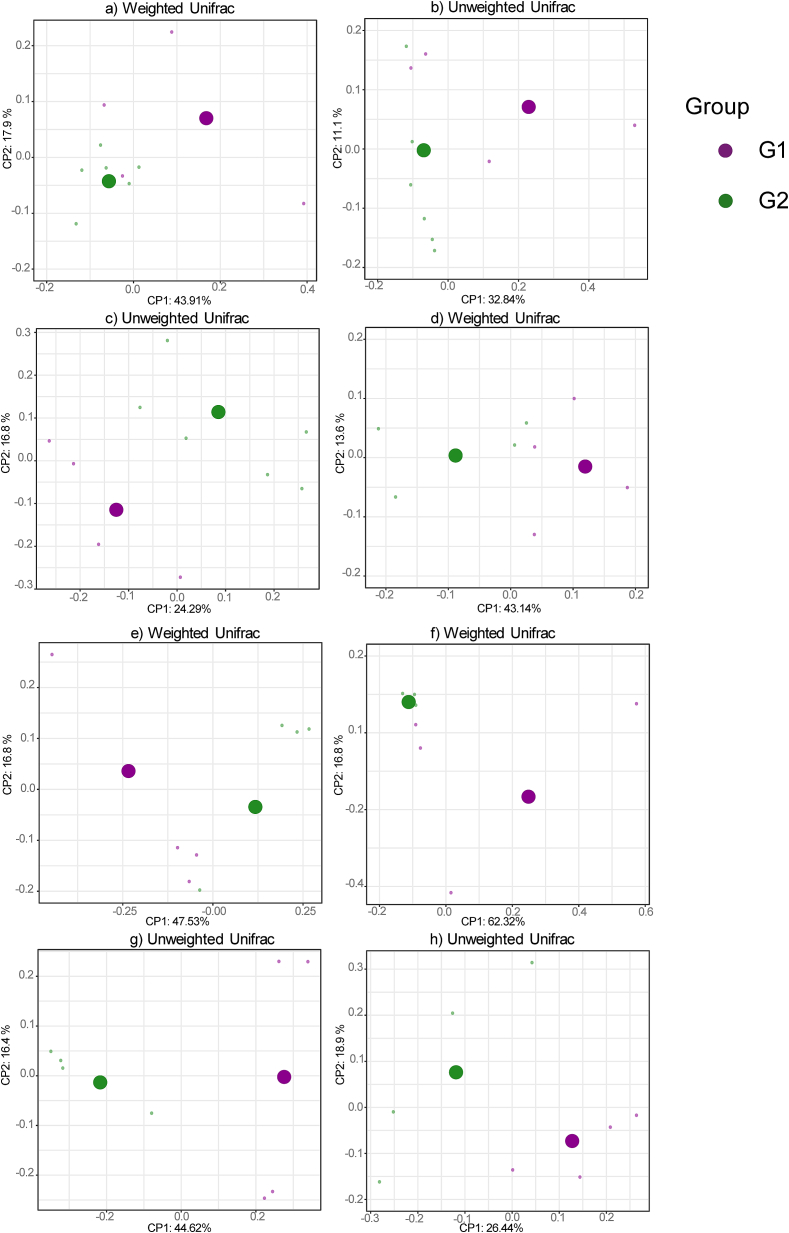
Significant differences (*P* < 0.05) in the beta diversity analysis in the comparison between G1 (abortion) and G2 (normal-delivery) within a herd depending on the sample. a) Faecal samples (herd B); b) Faecal samples (herd B); c) Nasal samples (herd B); d) Faeces samples (herd C); e) Milk samples (herd C); f) Blood samples (herd C); g) Vaginal samples (herd C); h) Nasal samples (herd C).

**Fig. 4:**
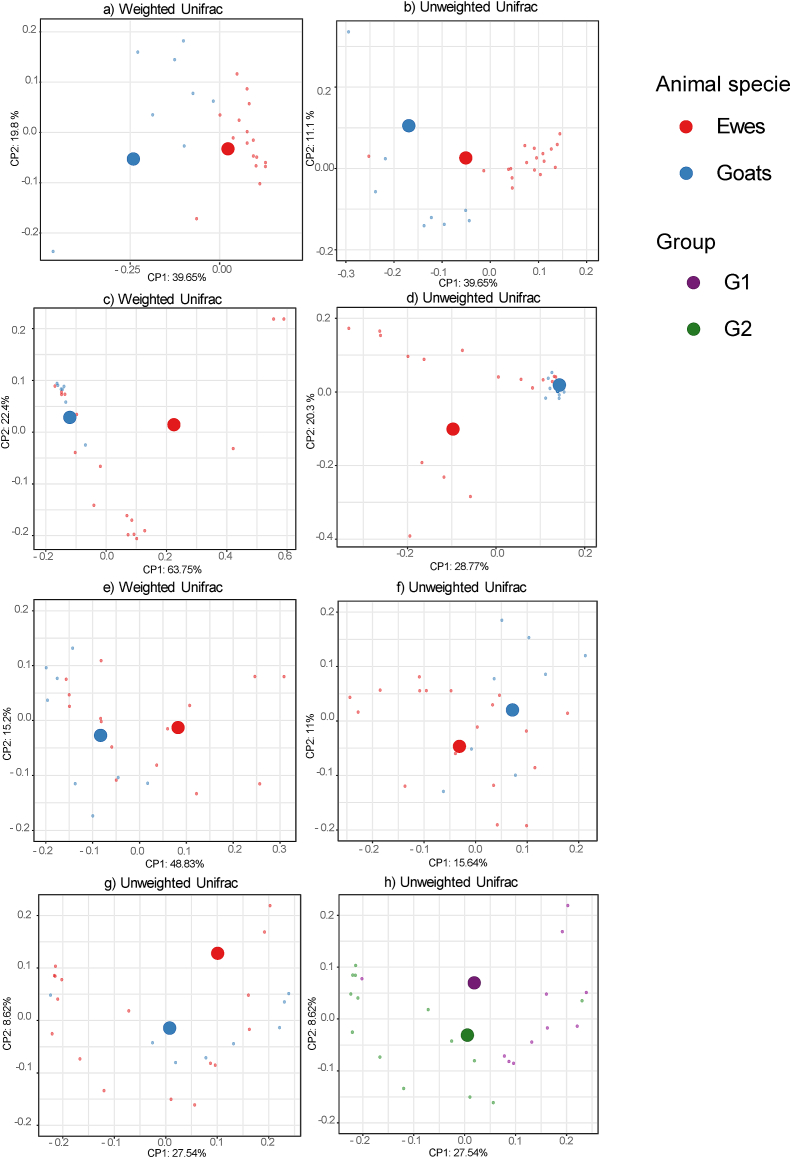
Significant differences in the beta diversity analysis in the comparison between goat vs. sheep (a,b,c,d,e,f,g) and females groups (G1, aborted vs. G2, normal-delivery; h) depending on the sample. a) Faecal samples; b) Faecal samples; c) Blood samples; d) Blood samples; e) Nasal samples; f) Nasal samples; g) Vaginal samples; h) Vaginal samples.

The results of Unweighted and Weighted UniFrac distances using the PERMANOVA test showed significant differences in the microbial community structure (*P* < 0.05) (Supplementary Table 3) between experimental groups within a herd (Fig. 3), species and experimental groups of all herds (Fig. 4). Significant results were observed in the dairy ovine herd (B) for faecal (Unweighted UniFrac and Weighted UniFrac, *P* < 0.05) and nasal samples (Unweighted Unifrac, *P* < 0.05) (Fig. 3a–c). In herd C (meat sheep), significant differences between G1 (aborted females) and G2 (normal-delivery females) for Weighted or Unweighted UniFrac (*P* < 0.05) were observed in all type of samples (Fig. 3d–h).

For animal species comparison, faeces, vaginal and nasal samples showed significant differences for Weighted or Unweighted UniFrac (*P* < 0.05) between sheep and goats (Fig. 4a–g). The comparison between the three aborted females' group (G1) and three normal-delivery females' group (G2) showed significant differences in vaginal samples (Unweighted Unifrac, *P* < 0.05) (Fig. 4h).

#### 3.2.3 General taxonomic composition: Phylum description

**Fig. 5:**
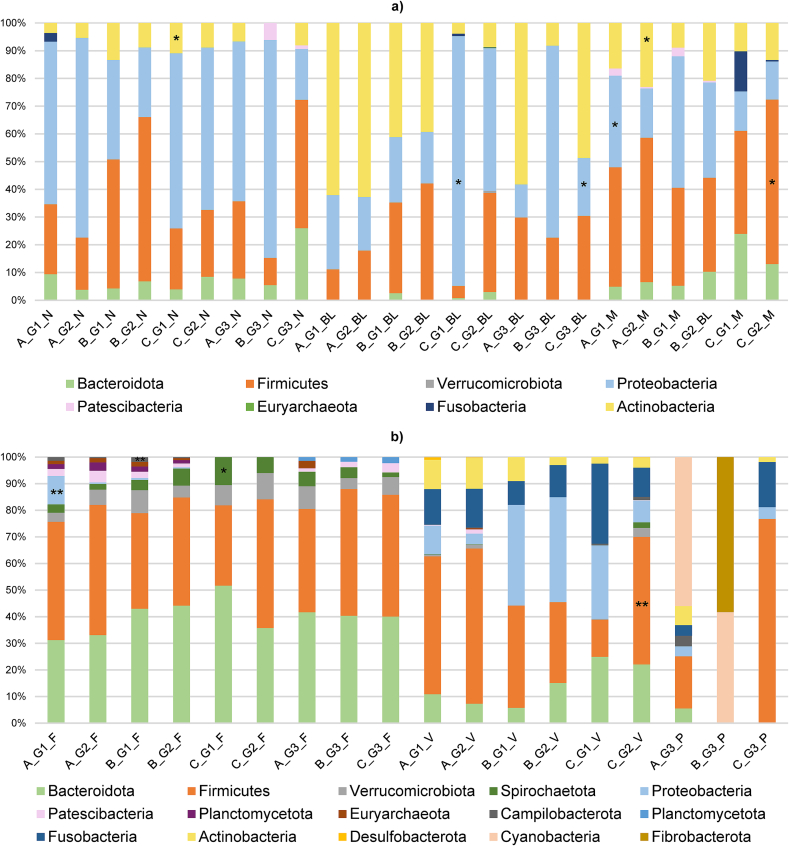
Relative abundance of taxa at the phylum level from each group of females (G1 and G2) and males (G3) from the three herds. Only the taxa with a mean relative abundance >1 % for the different samples are shown. A, herd A; G1, aborted females; N, nasal; B, herd B; G2, nomal-delivery females; C, herd C; G3, males; BL, blood; M, milk; F, faeces; V, vaginal; P, preputial. * *P*-value <0.05; ** *P*-value <0.01 (for females, the asterisk is placed on the on the experimental group (G1 or G2) with the significant higher relative abundance within a farm depending on the female's column means the higher relative abundance within a farm).

The phyla with the highest relative abundances (RA) from each group (G1: aborted females, G2: normal-delivery females, G3: males) and herd (A, B, C) depending on the type of samples are described in Fig. 5.

**Fig. 6:**
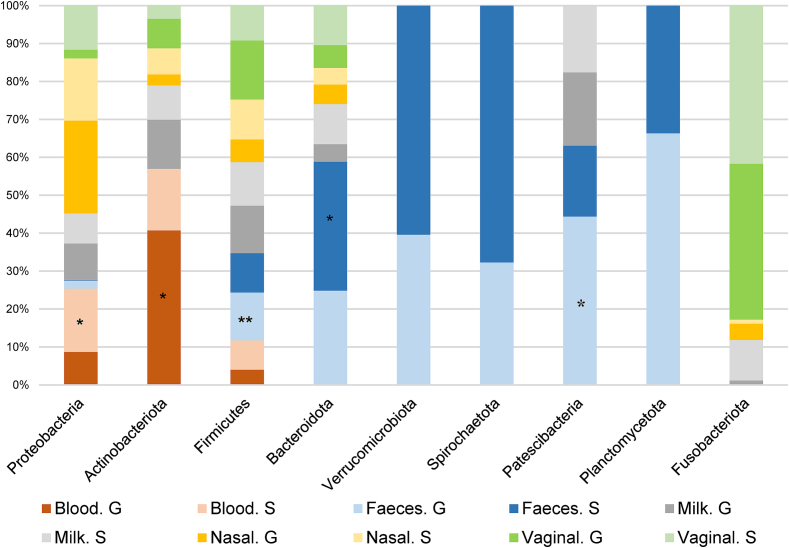
Relative abundance of taxa at the phylum level depending on the type of sample and animal species. Only the taxa with a mean relative abundance >1 % for the different samples are shown. G, goat; S, sheep. *P-value <0.05; **P-value <0.01 (the asterisk is placed on the type of sample from the animal species with the significant higher relative abundance).

The phylum description in the comparison between the three aborted (G1) and normal-delivery females (G2) groups is shown in Supplementary Fig. S1. Regarding the identified phyla in the different samples, Firmicutes had the highest RA (34 %), followed by Proteobacteria (29 %), Actinobacteriota (20 %), and Bacteroidota (16 %). Depending on the sample, the main phylum identified in nasopharyngeal samples was Proteobacteria for females (59 %) and males (50 %), with a significantly higher RA of Actinobacteria in aborted than in normal-delivery females from herd C (meat sheep). Actinobacteriota was the main phylum in goats from herd A (62 %), and from rams from herd B (40 %) and bucks (37 %) in blood samples. However, Proteobacteria was the most prevalent phylum (73 %) for herd C (meat sheep), and it was significantly higher for aborted females. For milk samples, Firmicutes was the most prevalent phylum in all herds (41 %), being statistically more prevalent in normal-delivery females in herd C (meat sheep). In herd A (goats), Proteobacteria (24 %) and Actinobacteria (19 %) showed significant differences (G1 > G2 and G1 < G2, respectively) for milk samples (Fig. 5a). Firmicutes was also the most abundant phylum in vaginal samples (41 %) across all three herds, in preputial samples (53 %) and was significantly higher in normal-delivery females (G2) vaginal samples of herd C (meat sheep). Although this phylum was one of the most abundant in faecal samples (29 %), Bacteroidota was the predominant phylum in the three herds (herd A = 31 %; herd B = 42 %; herd C = 6 %). In rams and buck's faeces, Firmicutes was the predominating phylum (42 %). In those samples, aborted females showed a significantly higher abundance of the phyla Proteobacteria in herd A (goats), Campylobacterota in herd B (dairy sheep), and Spirochaetota in herd C (meat sheep) (Fig. 5b). The phyla with the highest RA depending on the type of sample and the animal species are described in Fig. 6.

#### 3.2.4 General taxonomic composition: Genus description

**Fig. 7:**
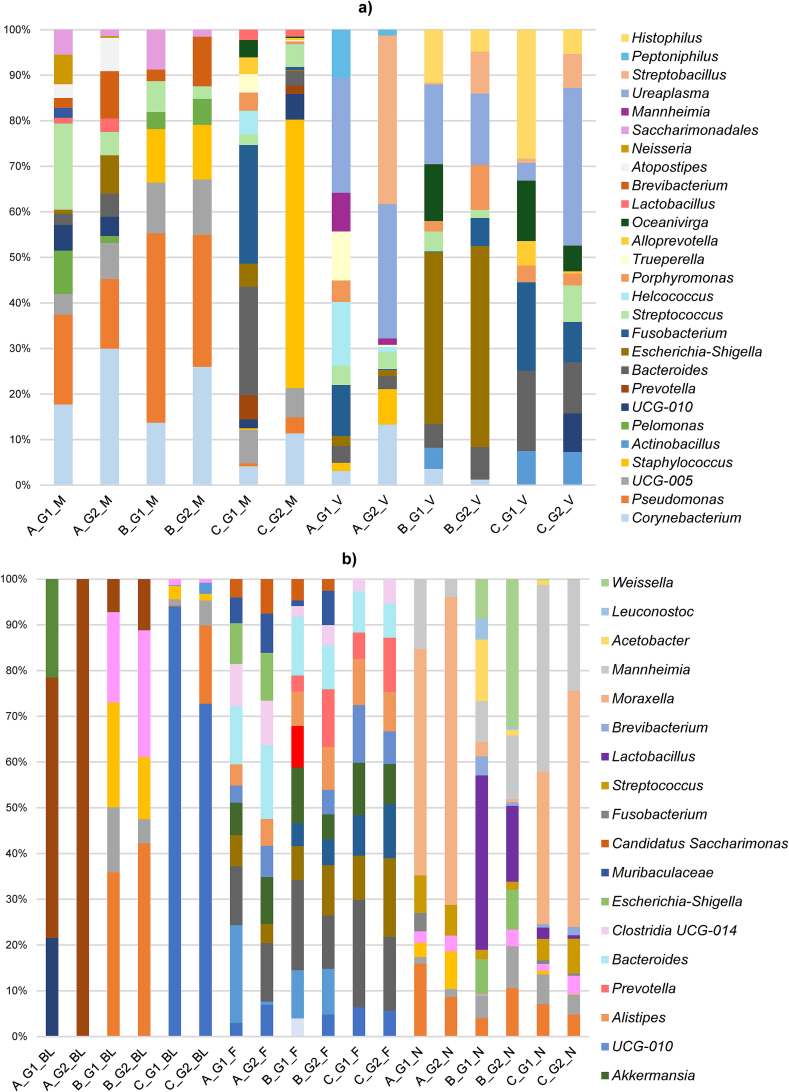
Relative abundance of taxa at the genus from each herd depending on the group (G1 or G2). Only the taxa with a mean relative abundance >2 % for the different samples are shown. A, herd A; G1, aborted females; M, milk; G2, normal-delivery females; B, herd B; C, herd C; V, vaginal; BL, blood; F, faeces; N, nasal.

**Fig. 8:**
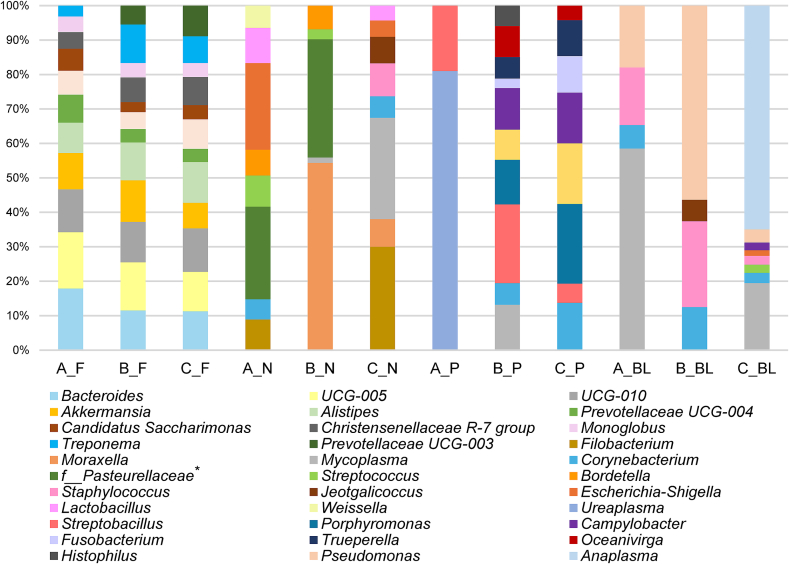
Relative abundance (> 2 %) of taxa at the genus level in male samples from each herd. Only the taxa with a mean relative abundance >1 % for the different samples are shown. *family taxa; A, herd A; F, faeces; B, herd B; C, herd C; N, nasal; P, preputial; BL, blood.

The most abundant genera from the three herds are described in Fig. 7 for females' samples, and in Fig. 8 for males' samples. Regarding the identified bacterial genera, nasal swabs were the samples that showed the most significant differences between the females' groups. *Moraxella* was the most abundant genus in nasal samples (24 %), followed by *Mannheimia* (11 %) and *Lactobacillus* (10 %) in the studied herds. In the case of aborted females, *Leuconostoc* and *Facklamia* in herd B (dairy sheep) and *Mannheimia* in herd C (meat sheep) showed a higher RA (*P* < 0.05). Other genera significantly higher in G2 (normal-delivery females) in nasal samples were *Acinetobacter* in herd A (goats), *Weissella* in herd B (dairy sheep) and *Staphylococcus*, *Salinicoccus*, *Citricoccus*, *Aerococcus* and *Treponema* in herd C (meat sheep) (*P* < 0.05). In the male nasopharyngeal samples, a genus from the family *Pasteurellaceae* (20 %) was the most abundant in bucks, and the second (27 %) in the rams from of the dairy herd (B). In bucks (herd A), the second most abundant genus was *Escherichia-Shigella* (19 %), followed by *Lactobacillus* (7 %). In herd B (dairy sheep), the main identified genus in rams was *Moraxella* (44 %), and in herd C (meat sheep) was *Filobacterium* (17 %), followed by *Mycoplasma* (17 %). This last-mentioned genus was the most abundant in blood samples from ewes (19 %) and rams (22 %) from herd B (dairy sheep), while *Staphylococcus* (12 %) in goats and *Pseudomonas* (11 %), and *Anaplasma* in females (68 %) and rams (50 %) in herd C (meat sheep). The main genera identified in milk samples were *Streptococcus* (5 %) in herd A (goats), *Pseudomonas* (10 %) in B, and *Staphylococcus* (10 %) in C, the last mentioned being higher in normal-delivery females (*P* < 0.01). For aborted females, the genus *Pelomonas* showed significant differences for herd A (goats), and *Bacteroides*, *Porphyromonas* and *Parviromonas* (*P* < 0.01) in herd C (meat sheep). Other genera with significant higher RA for G2 (normal-delivery females) in milk were *Corynebacterium*, *Salinicoccus*, *Pseudomonas* in herd C (meat sheep) (*P* < 0.05). Regarding vaginal samples, *Ureaplasma* (herd A = 17 %), *Escherichia-Shigella* (herd B = 28 %), and *Histophilus* (herd C = 13 %) were the most abundant genera. In herd C (meat sheep), *Jeotgalicoccus* and *Bacteroidales RF16 group* (*P* < 0.05) were significantly higher in the aborted females group. In normal-delivery females, *Staphylococcus* was significantly higher (*P* < 0.05) in herd A (goats). The most abundant genera for preputial swabs were *Ureaplasma* (71 %) in herd A (goats), *Streptobacillus* (14 %) in herd B (dairy sheep), and *Porphyromonas* (14 %) in herd C (meat sheep). Finally, in faecal samples, the genus *Rikenellaceae RC9 gut group* showed the highest RA in the three herds (7 %). On one hand, the aborted female's group from herd B (dairy sheep) showed a higher RA of the genus *Prevotellaceae UCG-001* and *Campylobacter* (*P* < 0.05), and the ones in herd C (meat sheep) were *UCG-010* and *Prevotellaceae UCG-004* (*P* < 0.05). On the other hand, *Alistipes*, *Prevotella* and *Muribaculaceae* for herd B (dairy sheep), and *Treponema*, *Prevotellaceae UCG-003* (*P* < 0.05) for herd C (meat sheep) were higher for normal-delivery females. In males' faecal samples, *Bacteroides* (9 %) in herd A (goats), *UCG-005* (6 %) in herd B (dairy sheep), and *UCG-010* (6 %) in herd C (meat sheep), were the most abundant genera.

#### 3.2.5 General taxonomic composition: Species description

**Fig. 9:**
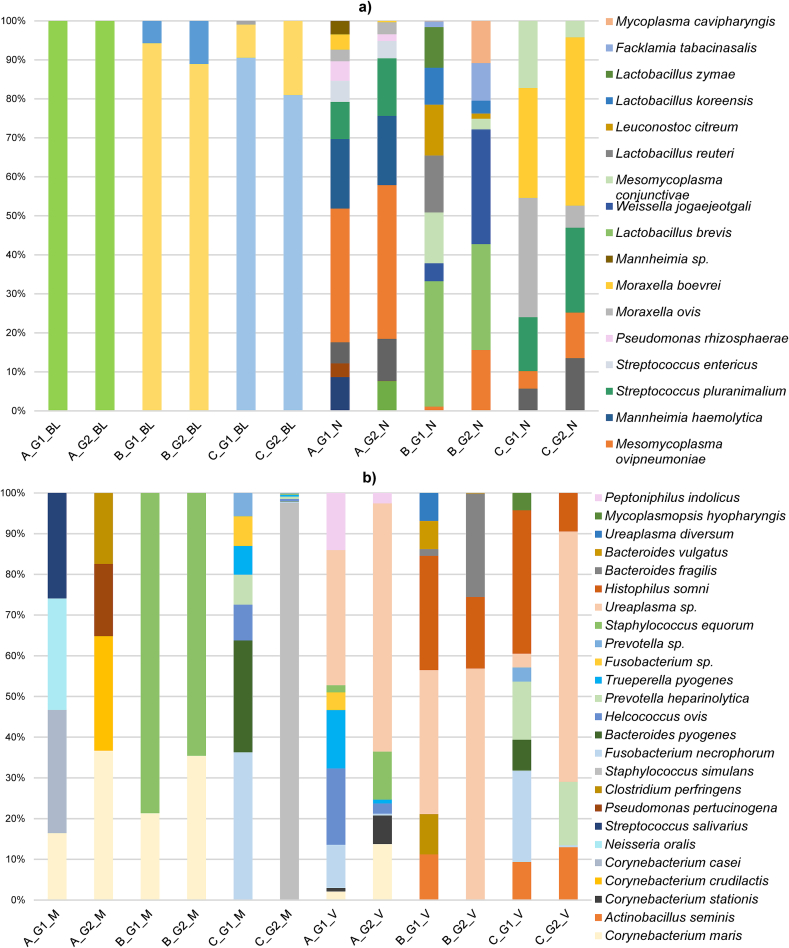
Relative abundance of taxa at the species level from each herd depending on the group (G1 or G2). Only the taxa with a mean relative abundance >1 % for the different samples are shown. A, herd A; G1, aborted females; BL, blood; G2, normal-delivery females; C, herd C; N, nasal; M, milk; B, herd B; V, vaginal.

**Fig. 10:**
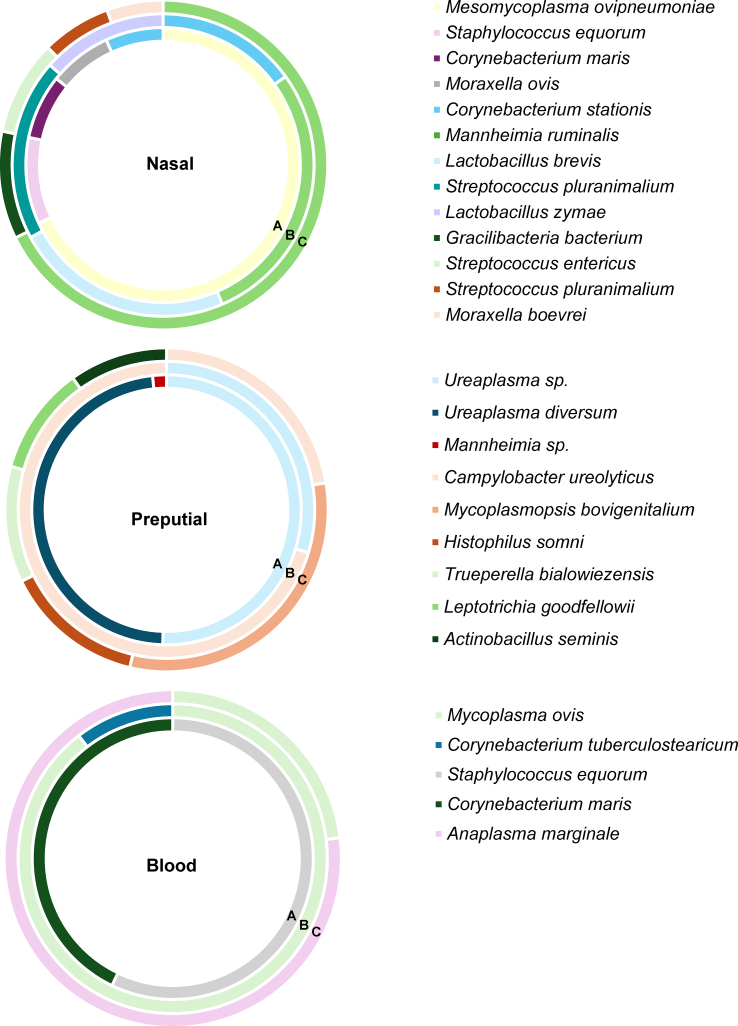
Relative abundance of taxa at the specie level from each group of males from the three herds. Only the taxa with a mean relative abundance >1 % for the different samples are shown. A, herd A; B, herd B; C, herd C.

Taxonomic diversity analysis did not identify any species with a RA greater than 1 % in faecal samples from the three herds. The most abundant species from female samples from the studied herds are described in Fig. 9, and Fig. 10 for male samples. The metagenomic analysis described the most abundant species identified in nasopharyngeal samples as *Mesomycoplasma ovipneumoniae* (9 %) in herd A (goats)*, Lactobacillus (L.) brevis* (5 %) in herd B (dairy sheep), and *Moraxella boevrei* (5 %) in herd C (meat sheep). Significant differences were identified in herd B for the species *Leuconostoc citreum,* higher in aborted females (*P* < 0.05), and the species *Facklamia tabacinasalis* and *Mycoplasma cavipharyngis* for normal-delivery females (G2) (*P* < 0.05). In herd C (meat sheep), *Moraxella ovis* was significantly higher in aborted females (*P* < 0.01) and *Staphylococcus equorum* in G2 (*P* < 0.05). Nasopharyngeal samples showed the highest RA of species from the *Lactobacillus* genus, in females and males, although this abundance was lower than 1 %. The identified species, from highest to lowest abundance, were *Streptococcus (S.) salivarius*, *L. brevis*, *L. reuteri*, *L. koreensis* and *L. zymae*. In males' nasopharyngeal samples, *Mesomycoplasma ovipneumoniae* (herd A = 17 %) and *Mannheimia ruminalis* (herd B = 2 %; herd C = 23 %) were the most abundant species. Regarding blood samples, the species with the highest abundance were *Curvibacter gracilis* for females (1 %) and *Staphylococcus equorum* (2 %) for bucks in herd A, *Mycoplasma (M.) ovis* (females = 19 %; rams = 22 %) in herd B (dairy sheep), and *Anaplasma (A.) marginale* (females = 60 %; rams = 50 %) in herd C (meat sheep). In milk samples, *Corynebacterium stationis* (herd A = 1 %), *Staphylococcus equorum* (herd B = 2 %), and *Staphylococcus simulans* (herd C = 10 %) were the most abundant species. For vaginal samples, *Ureaplasma* sp. (herd A = 17 %), *Prevotella heparinolytica* (herd B = 15 %), and *Histophilus somni* (herd C = 13 %) were the species with the highest RA. Preputial swab samples indicated *Ureaplasma* sp. (herd A = 36 %), *Campylobacter ureolyticus* (herd B = 8 %), and *Mycoplasmopsis bovigenitalium* (herd C = 9 %) as the main species present. Taxonomic analysis in herd A (goats) identified the genus *Coxiella* and *Cb* in vaginal samples from three q-PCR positive goats (RA < 1 %; one from the aborted (G1) and two from normal-delivery group (G2). In dairy sheep from herd B, the genus *Coxiella* was identified in vaginal samples from a negative q-PCR normal-delivery sheep and *Cb* in milk from an aborted ewe (RA < 1 %) (q-PCR negative). The significant taxa (*P* < 0.05) for each type of sample and herd are described in Supplementary Table S4, and for the species comparison (sheep/goats) in Supplementary Table S5.

#### 3.2.6 General taxonomic composition: Environmental description

**Fig. 11:**
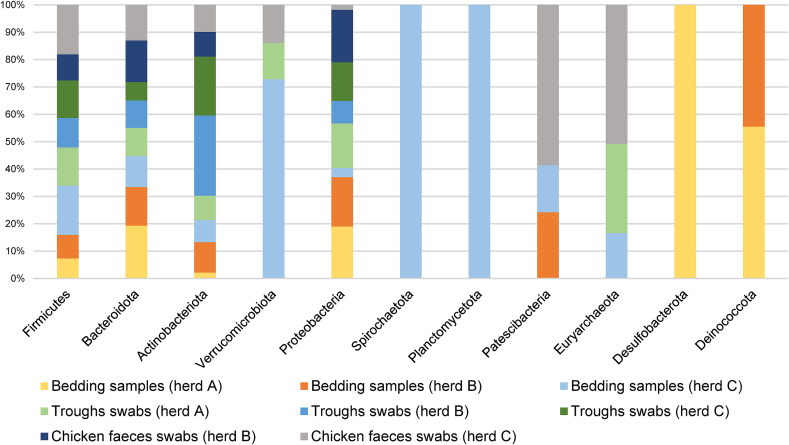
Relative abundance of taxa at the phylum level from each type of environmental samples from the three herds. Only the taxa with a mean relative abundance >1 % for the different samples are shown.

Regarding environmental samples, Firmicutes (30 %), Bacteroidota (29 %) and Proteobacteria (18 %) were the most abundant phyla. Firmicutes was the most abundant in the bedding samples (43 %) of the meat sheep herd (C), domestic samples (43 %), and in trough swabs (33 %) and for goats from herd A (33 %) too. Bacteroidota was the most abundant phylum in the bedding samples of herd A (45 %) and in the bedding (33 %) and domestic samples (35 %) of herd B (dairy sheep). Actinobacteriota was the most prevalent phylum in the trough sample of herd B (36 %). Results of genus and species taxa in the three types of environmental samples are described in Table 5. In the taxonomic analysis at the species level, the species *Cb* was detected in the bedding samples and troughs' swabs of the goat herd (RA < 1 %). Bacterial phyla with the highest RA are shown in Fig. 11.

**Table 5:** Taxonomic description (genus and species) for environmental samples and their relative abundance (%).

		Genus	Species
Bedding samples	Herd A^1^	*Ulvibacter* (23)	*Pseudomonas pertucinogena* (3)
		family *Balneolaceae* (7)	*Acholeplasma axanthum* (2)
		*Taibaiella* (5)	*Luteimonas* sp. (2)
		*Pusillimonas* (5)	
	Herd B^2^	*Corynebacterium* (8)	*Luteimonas* sp. (3)
		*Halomonas* (7)	*Pseudomonas pertucinogena* (2)
		*Myroides* (5)	*Corynebacterium stationis* (1)
		*Oceanobacillus* (4)	
	Herd C^3^	*Corynebacterium* (8)	*Corynebacterium maris* (3)
		*UCG-005* (4)	*Facklamia tabacinasalis* (1)
		*Bacteroides* (4)	*Luteimonas* sp. (1)
		*Rikenellaceae RC9 gut group* (4)	
Troughs samples	Herd A^1^	*Acinetobacter* (7)	*Acinetobacter lwoffii* (5)
		*Desemzia* (5)	*Corynebacteriu maris* (2)
		*Corynebacterium* (5)	*Staphylococcus equorum* (1)
		*Flavobacterium* (4)	
	Herd B^2^	*Sphingobacterium* (18)	*Corynebacterium variabile* (6)
		*Corynebacterium* (9)	*Weissella jogaejeotgali* (5)
		*Brevibacterium* (7)	*Paracoccus alcaliphilus* (3)
		*Weissella* (6)	
	Herd C^3^	*Staphylococcus* (21)	*Kocuria* sp. (2)
		*Sphingobacterium* (9)	*Weissella jogaejeotgali* (1)
		*Brevibacterium (9)*	*Corynebacteriu maris* (1)
		Weissella (3)	
Domestic animal samples^4^	Herd B^2^	*Myroides* (9)	*Acinetobacter lwoffii* (4)
		*Ulvibacter* (6)	*Ignatzschineria* sp. (3)
		*Sphingobacterium* (5)	*Luteimonas* sp. (3)
		*Lactobacillus* (3)	
	Herd C^3^	*Bacteroide*s (14)	*Olsenella* sp. (5)
		*Olsenella* (6)	*Candidatus saccharibacteria* (2)
		*Ruminococcus torques group* (5)	*Acinetobacter lwoffii* (1)
		*Lactobacillus* (4)	

1 Goats.

2 Dairy sheep.

3 Meat sheep.

4 Faecal samples.

## 4 Discussion

The present study reports for the first time the significant relationship between alterations in the microbiota of goats and sheep present in *Cb*-circulating flocks and the female's health status (aborted/ normal-delivery). This pathogen was only detected in a low RA (< 1 %) in one milk sample, in four vaginal samples and in one environmental sample by metabarcoding. Other abortive pathogens, including *L. monocytogenes* and *C. abortus*, were not detected in either the animals or the environment. We hypothesize that although *Cb* is not abundant in the microbiota of infected hosts in clinically affected flocks, its presence could cause disorders in the general and local microbiota of the animals that lead to different courses of infection and disease. The presence of *Cb* in the nasopharynx of goats, previously unreported, suggests a possible respiratory tropism in this species. Our study is the first to analyze the microbiota of anatomical sites as the mammary gland, respiratory, digestive and genital tracts and blood in small ruminants. New bacterial species not previously described in small ruminants are reported, as well as new tropisms of other already-known species.

### 4.1 Different herd infection rates, first detections of *Cb* in the caprine nasopharyngeal cavity, and the importance of implementing collective control measures

This study confirms the circulation of *Cb* in dairy and meat sheep and goat herds in the eastern Iberian Peninsula showing a seropositivity of 100 % in goats and 68 % in sheep, consistent with other studies that reported a seropositivity of 70 % in sheep [58] and 90 % in goats [59].

Regarding the analysis techniques, diagnosis by q-PCR allowed the *Cb* detection in different goat samples. Nevertheless, in the case of sheep, *Cb* was detected only in three vaginal samples from dairy sheep (herd B). The vaginal route was suggested as the most relevant in sheep [14]. However, our results suggest that it could be necessary to obtain a high number of vaginal samples to confirm the diagnosis by q-PCR in ovine flocks. Concerning shedding animals, one of these vagina q-PCR positive aborted sheep (G1) was seronegative. It is known that the serological and excretory status of *Cb* in an animal do not necessarily correlate [15,60]. In our study, one goat and eleven sheep were ELISA positive and q-PCR negative for all samples, consistent with the low specificity observed (30 %) in validating serology against the gold standard (q-PCR) (Table 3). Therefore, seropositivity may not be indicative of recent *Cb* excretion, suggesting that the use of serology as the only diagnostic method to assess individual health status in sheep may not be appropriate for Q fever [15,58,61,62].

The anatomic location where *Cb* was most frequently detected in goats were in the deep vagina and nasopharynx, followed by faeces. Thus far, the bibliography describes the vaginal, faecal, and milk as the main shedding routes of *Cb* in goats [12,59]. Only one sample of raw milk from a goat was positive in our study. The anecdotal excretion in milk observed in our study could indicate that colonization of the udder is not always common in small ruminants. About shedding routes, more than 80 % of the goats tested positive for *Cb* in the nasopharyngeal sample in herd A (Table 2). Although this is the first report on goats, these samples have been described casually for *Cb* detection in sheep [63]. It is known that the inhalation of the bacteria is the main route of transmission between humans and animals [64]. Anecdotally after the present study, more than 30 goats died in herd A and *Cb* detection was confirmed in nasal and lung swabs from a dead goat [65]. Further studies are necessary to evaluate the potential use of this sample for Q fever diagnosis and the importance of a possible respiratory tropism in small ruminants. Nasopharyngeal swabs evidenced a notable agreement with vaginal swabs and faeces, justifying the combination of the samples to improve the specificity of the diagnosis. The infection of the studied animals (seropositivity or detection by q-PCR) did not show a significant correlation with clinical signs (abortion or normal delivery), consistent with other descriptions in natural [4] or experimental conditions [66]. Seropositive rams (50 %; 2/4) and bucks (100 %; 2/2) were detected in the three herds. Additionally, the nasopharyngeal sample of one seropositive buck tested positive, highlighting the potential risk of *Cb* transmission through nasal secretions during natural mating, which has not been considered until now. The risk of venereal transmission should not be overlooked either, as rams with foreskins [67] and semen [20] contaminated with *Cb* have also been reported.

### 4.2 Trough contamination by *Cb* has not been previously considered

In the present study, *Cb* was detected in bedding samples by q-PCR from herd A (goats) and B. This has been reported in other studies [68], where authors detected *Cb* DNA in these samples for one year [21]. However, trough samples from the lambing or kidding areas had not been considered until now as potential contamination and transmission sources for animals, even though it is not usually disinfected or cleaned in Q fever outbreaks. In our study, herd A tested positive for q-PCR of this type of sample, which could be related to the high pathogen detection frequency in nasal samples. Cleaning and even disinfection of troughs should be implemented as a Q fever's biosecurity measure.

### 4.3 The microbiota of aborted and normal-delivery sheep was discordant, unlike that of goats

Alpha diversity analyses also showed significant variations in the number (richness) and abundance (evenness) of microorganisms between aborted and normal-delivery group from both sheep herds (B and C), unlike goats (A). In both ovine herds, the aborted group (G1) presented a significantly higher evenness (Pielou's index) in faecal samples than normal-delivery females (G2) (Fig. 1). In herd C (meat sheep), G2 showed a higher richness for blood and faecal samples, and the beta diversity analyses revealed that the groups of females from this herd were the most dissimilar among the study in faecal, milk, nasal, blood, and vaginal samples, although. This herd had the lowest rates of abortions and seropositive animals, with no *Cb* detected in any sample. While a similar situation may apply to herd B (dairy sheep), the opposite occurred between groups of goats in herd A. In this flock, females from both experimental groups exhibited a similar high frequence of *Cb* detection and seropositive results. Microbial diversity between aborted and non-aborted female small ruminants has not been previously evaluated. Differences may result from animal species influence [69], or infection levels among herds. This leads us to hypothesize that the existence of a common microbiota in the flock could reflect an imbalance in the overall microbiota, characterized by the absence of groups of animals with a protective microbiota, ultimately leading to a higher rate of infection and clinical symptoms. Other factors that could impact microbial diversity between females, such as climate, environmental pollution, and geographical distribution, should also be considered [70]. Therefore, longitudinal studies are needed to evaluate and compare the microbiota of small ruminants [24].

### 4.4 First evidence of *Cb* negative impact on general and vaginal microbiota

In the vaginal samples comparison between the three aborted (G1) and normal-delivery (G2) groups, normal-delivery females showed a higher richness and different microbial composition than G1 (aborted females). This result indicates that *Cb*-aborted goats and sheep have a lower number of observed ASVs in vaginal microbial, as well as significant bacterial phylogenetic differences compared to females with normal delivery. A recent study found that vaginal community richness, evenness, and diversity decreases at the time of pregnancy diagnosis, especially in non-pregnant ewes [27]. The potential impact of the reproductive system microbiome on *Cb*, and vice versa, should encourage further studies.

Depending on the animal species, faeces, nasal and blood samples showed a higher alpha diversity in sheep (Fig. 2) and differences in beta diversity compared to goats (Fig. 4). Regarding taxonomic diversity, there were variations in the number of detected phylotypes depending on the herd and the type of sample (Supplementary Table S1). The goat herd showed the fewest identified phylotypes, which is in accordance with the lowest richness mentioned before. Of the three flocks, the goat flock showed more severe clinical symptoms along with a higher frequency of *Cb* detection. It should also be considered that sheep are less susceptible to contracting Q fever than goats [69], and that in semi-extensive herds the *Cb*-circulation is lower [71]. In addition, the higher frequency of bacteria with antimicrobial potential in the microbiota of small and extensive ruminants has also been suggested due to less exposure to antibiotics [72].

### 4.5 Vaginal dysbiosis linked to abortion shows increased bacterial genera associated with reproductive failure

Significant differences in certain bacterial genera were observed in the vagina and foreskin of the studied herds. Regarding the genera significantly more abundant in normal-delivery females (G2), *Streptobacillus* was one of them in the vaginal samples in all herds, it was also identified in preputial samples in the dairy sheep herd (B). This bacterial genus has been associated with the vaginal microbiota of healthy sheep [73,74]. Similar results were observed for *Staphylococcus*, one of the most prevalent genera in the vagina of goats, more prevalent in pregnant than non-pregnant ewes [75], which shows a drastic decrease as gestation progresses in sheep [76]. This genus had a significantly higher abundance in the vagina of goats from the G2 group (normal-delivery females) in herd A.

On the other hand, *Ureaplasma* showed the highest RA in the herds with high abortion rates (herds A and B) in vaginal samples and the prepuce sample of one buck. This bacterium has been associated with reproductive failures, abnormal spermatic morphology, clumping, venereal transmission, infertility and low birth weights and decreased milk production in domestic ruminants [[77], [78], [79], [80]]. Moreover, the influence of this genus and its species on the reproductive microbiota of small ruminants is uncertain, since it has also been linked to higher pregnancy rates [27,32] and detected in ejaculates [29] and foreskins of asymptomatic males [27]. *Histophilus* was one of the most abundant genera in ovine vaginal samples. This genus was described as significantly lower in vaginal samples from pregnant ewes [32]. In sheep vaginal samples, one of the main abundant species was *Histophilus (H.) somni* which had greater RA in G1 (aborted females) of both ovine herds. This result is coherent with previous results where this species was more frequent in non-pregnant vaginal samples from ewes and linked to reproductive disorders [32,76,81]. In addition, our results identified *Actinobacillus* in a higher abundance in G1 (aborted females) in both ovine flocks. *Actinobacillus (A.) seminis* was detected in samples from the vagina and blood of ewes, being particularly higher in G1, and in ram's preputial samples. This species has been associated with abnormal semen quality and reproductive disorders in rams and ewes [82,83]. Serrano et al. [32] identified *A. seminis* in ewes' vaginal samples from herds with high artificial insemination failure rates and in rams' preputial swabs. The genus *Jeotgalicoccus* was significantly higher in the aborted vaginal samples from herd C (meat sheep). However, it has been described as part of the normal vaginal microbiota of nulliparous ewes [27]. Other previously described genera in vaginal samples, from ewes that failed to get pregnant, include *Facklamia*, *Corynebacterium* and *Fusobacterium* [76]. In our study, the genus *Fusobacterium* was found to be more abundant in aborted females' vaginal, milk, and nasal samples. This genus has been described as part of the normal ovine vaginal microbiota [32,84], although members of the *Fusobacterium* genus are known for contributing to bacterial vaginosis, abortions and premature births in ruminants [76,85]. In preputial samples, *Corynebacterium* and *Fusobacterium* were the main genera identified, consistent with previous findings [27]. Some species identified in vaginal samples included *Corynebacterium maris*, as well as *Corynebacterium tuberculostearicum*, species not described before in this type of sample from sheep and goats. This genus has been described by authors as a possible bacteria associated with bacterial vaginosis and pregnancy outcomes [32,76]. Despite reports of the normal presence of *Mycoplasma* in the vaginal microbiota of sheep [28], our vaginal microbiota results did not show a predominance of this genus that could indicate a reproductive tropism. Despite this, this genus was indeed identified in ram's preputial samples from herd B (dairy sheep), as previous authors describe in ovine [86]. *Mycoplasmopsis hyopharyngis*, a traditionally porcine species of the upper respiratory tract [87], was identified for the first time in the aborted females from herd C (meat sheep). In our opinion, the presence of respiratory-trophic bacterial species in other locations may result from vaginal contamination with faeces containing swallowed nasopharyngeal mucus or by direct contact between the nasal mucus and vulva of animals. *Mycoplasmopsis bovigenitalium*, which is one of the main agents described in the preputial microbiota of bulls [88] and related to infertility in cows [89], was identified in ram's preputial swabs, and in G1 (aborted females) a with low RA (< 1 %) in vaginal samples from herd B (dairy sheep). Although not generally linked to reproductive failure, *Mannheimia* was identified in greater abundance in G1 (aborted females) in vaginal and nasal samples and *Streptococcus* was in higher abundance in the vagina of flocks A and B in G1. Consequently, abortion could trigger factors that positively influence the viability of these bacterial populations in herds where *Cb* circulates, or vice versa. The effect of dysbiosis on the female's reproductive success is assumed in ruminants [90]. Future studies should clarify whether the increase in vaginal bacterial populations linked to reproductive failure is associated with infertility in ruminant herds with Q fever. Although little is known so far about future infertility in herds of ruminants *Cb*-infected small ruminants, cases of infertility/subfertility associated with dairy cattle with Q fever have already been observed [91]. In our opinion, the changes in the vaginal microbiota are a consequence of the pathological condition of the animals. On the other hand, bacteria observed in the vaginal and preputial microbiota in our results (*Ureaplasma* spp., *H. somni*, *A. seminis*, *Corynebacterium* spp., *Fusobacterium and Mycoplasmas* spp.) support Barba et al. [27] theory of bacterial modulation through natural mating.

### 4.6 Different milk microbiota patterns were observed between female groups, particularly in the least affected herd (C)

Statistical differences in milk samples were observed in herd C (meat sheep) for both female groups. Again, the less affected and infected flock showed different microbiota patterns between groups of normal-delivery and aborted females. *Porphyromonas* was found to be significantly higher in G1 (aborted females). It was described in human milk as a biomarker of digestive, inflammatory, and metabolic disorders in women [92]. Another significant genus for G1 was *Bacteroides* in herd C (meat sheep), previously described in healthy goat milk [24]. Regarding normal-delivery females milk samples, *Corynebacterium* was also significantly higher compared to G1 (aborted females), including the species *Corynebacterium crudilactis* and *Corynebacterium casei* which have been reported in dairy cow's products [93,94]. Still within herd C, the genus *Salinicoccus* and *Staphylococcus*, described in sheep-healthy milk [26,38,95], were significantly higher in milk from females of G2 (normal-delivery females). Regarding this last genus, *Staphylococcus simulans* and *Staphylococcus equorum* were identified in sheep samples, both species have been described in ruminants with subclinical mastitis [96], and in caprine raw milk [24]. The viability of the genera *Corynebacterium*, *Salinicoccus* and *Staphylococcus* in the mammary gland could be negatively affected by the health status in herds where *Cb* is circulating. We should not rule out either that certain bacterial populations counteract the effects of *Cb* infection.

### 4.7 The nasopharyngeal microbiota of normal-delivery females showed increased levels of specific bacterial populations in sheep herds

Contrary to what was observed in sheep flocks, the respiratory microbiota of the goat flock was uniform among groups of females. Certain genera were statistically significantly higher in G1 (aborted females), such as lactic acid bacteria (LAB) species like *Facklamia* and *Leuconostoc* (herd B), the latter having been described in ruminant's raw milk [97]. Particularly, *Leuconostoc citreum*, which was significantly higher in G1 (aborted females) from herd B (dairy sheep), has been described as a potential *in vitro* antimicrobial in humans [98]. *Mycoplasma cavipharyngis*, a species described as a pneumonia pathogen [99] and reported for the first time in small ruminants in this study, was significantly higher in nasal swabs at herd B (dairy sheep) for normal-delivery females (G2). In G2, LAB species such as *Weissella* (herd B) and *Aerococcus* (herd C; meat sheep) were statistically higher, as well as other bacterial genera such as *Salinicoccus*, *Citricoccus*. These last three genera have been described in the normal milk microbiota of domestic ruminants [38,[100], [101], [102]]. *Acinetobacter*, *Staphylococcus*, and *Treponema* were also statistically significant higher for normal-delivery females from herd C (meat sheep). *Acinetobacter* has been found in raw milk and meat from livestock [103]. In this study, *Acinetobacter lwoffii,* previously reported in bovine vaginal samples during abortion episodes [104], was identified in sheep samples. At last, *Treponema* had a significantly higher abundance in normal-delivery females' nasal and faecal samples. This opportunistic pathogen is linked to contagious digital dermatitis in bovine and ovine foot tissues and is present in faecal samples of stressed pigs [105,106].

### 4.8 Differences in the faecal microbiota between aborted and normal-delivery females

In faecal samples, taxonomic genera from the family *Prevotellaceae* and *Rikenellaceae*, and order *Bacteroidales* showed the highest RA and differences between sheep female groups which is similar to previous small ruminant's faecal microbiota studies [107,108]. Specifically, *Alistipes* was more abundant (*P* < 0.05) in normal-delivery females from herd B (dairy sheep). Koester et al. [76] pointed out the genus *Alistipes* as another beneficial genus for gestation in sheep and it was also described in the cow's vaginal microbiota [109].

### 4.9 Firmicutes as the most relevant phylum in the microbiota of several locations: The environmental microbiota as a bacterial axis

Regarding the presence of *Cb* in environmental samples by metabarcoding, it was detected only in bedding samples from herd A. These data provide a new perspective on *Cb* environmental contamination in small ruminant herds, suggesting a potentially lower contamination load than expected. However, the presence of the bacteria is already an important risk factor for the human population due to the pathogenicity, high persistence, and its ability to spread [2].

Firmicutes was found to be the most abundant phylum in the analyzed environmental samples. This phylum was also one of the most abundant in vaginal, preputial, milk and faeces samples. Indeed, numerous bacterial species and genera were present in several sites including faeces, although they were not classically linked to their tropism. In cattle, initial studies on the gut-lung axis reveal emerging insights into the interaction between microbiota from different anatomical locations [33,110]. We propose that in addition to the above-mentioned interactions between respiratory, digestive and genital microbiota via mucus ingestion and excretion, possible contributions from the environmental microbiota should also be considered. The influence of bacterial communities in the litter on the vaginal microbiota has been suggested [27] and may also extend to the prepuce and mammary gland via the teat canal. Based on this, it is not unreasonable to think that this general abundance of Firmicutes could be a first indication of the existence of an axis between the microbiota of different anatomical locations in small ruminants not contemplated to date, which could even include the one present in the environment. Firmicutes could influence the modulation of the health of these animal species, especially considering that this phylum includes both LAB species with reported antimicrobial activity against small ruminant pathogens [72,111].

### 4.10 The abundance of *Lactobacillus* in nasopharynx may reflect an as-yet-unknown role in the respiratory microbiota

*Lactobacillus* is one of the main genera described in healthy milk [24,26] and faeces from sheep [25]. On the contrary, low abundances of *Lactobacillus* spp. in ovine vaginal samples have been described [84,112]. Although they are not abundant, their presence in the global microbiota of small ruminants could be key to preventing the proliferation of pathogens. Among other functions, they can maintain, for example, pH values  that are not favourable for certain pathogens [27]. In the present study, the genus *Lactobacillus* showed a low abundance (< 1 %) in the samples analyzed, except for nasopharyngeal swabs in both sexes. The role of this genus in the respiratory tract of small ruminants has not yet been studied. Some *Lactobacillus* species were identified in female and male nasal samples. It is the case of *L. reuteri*, which is common in the gut microbiota of warm-blooded animals [113] and is used as a probiotic for respiratory disease in humans [114]. Moreover, *L. brevis*, one of the most common probiotics in milk of ruminants [115], and finally *L*. *koreensis* and *L. zymae,* previously isolated from fermented foods [113,114], were also identified. Considering the ability of probiotics to indirectly influence the microbiome composition and improve animal health [116,117], there may be the possibility of developing studies to evaluate the use of *Lactobacillus* as an alternative to antibiotics for the control or prevention of respiratory diseases in small ruminants.

## 5 Conclusion

The results of this study show that the clinical severity of abortion outbreaks can be associated with higher and lower infection rates and Q fever control and prevention strategies should be implemented throughout the entire herd. Cleaning and disinfection of troughs should also be included in the biosecurity protocols for herds with Q fever due to the potential for contamination. Regarding diagnosis, studies based only on serological diagnosis would not reflect the real epidemiological situation of *Cb* infections. Nasopharyngeal swab sampling for q-PCR could improve the sensitivity of Q fever diagnosis in goat herds. Moreover, the respiratory tropism of *Cb* and its consequences could be underestimated in small ruminants. Our results show a global deterioration of the microbiota in herds with severe infection never contemplated until now. Furthermore, the dysbiosis observed in this study on local bacterial populations linked to abortion suggests the need for further studies to assess the likely consequences on milk production and quality, fertility or the respiratory tract. The study of the microbiota present in different anatomical locations of the same animal and its relationship with the host allows us to obtain a novel approach in the study of important ovine and caprine zoonotic diseases such as Q fever.

## Funding

This work was funded by the Spanish AEI-MICINN (PID2020-119462RA-I00/AEI/10.13039/501100011033; PI ÁG-M). The publication is part of the grant PID2023-152404OB-I00, funded by MCIU/AEI10.13039/501100011033 and FSE+ (PI ÁG-M) and financing UCH-CEU (PI ÁG-M) aid for Recognized Research Groups (GIR23/27), the Consolidation of Research Indicators (INDI23/27), a grant PID2022-137961OB-I00 (JQ) funded by MICIU/AEI/ 10.13039/501100011033/ERDF/EU, and a Development and Innovation (I + D + i) contract (UCH-CEU and CEVA Santé Animale, PI: ÁG-M). RT-P is supported by a pre-doctoral contract FPI of the Generalitat Valenciana (CIACIF/2021/245). MT is the recipient of a pre-doctoral contract (FPI) by the CEU-UCH. ÁG-M and JQ are supported by “Ramón y Cajal” contracts of the Spanish Ministry of Science, Innovation and Universities (RYC2021-032245-I and RYC-2018-024985-I) funded by MICIU/AEI/10.13039/501100011033 by ESF Investing in your future.

## Declaration of generative AI and AI-assisted technologies in the writing process

During the preparation of this work the author(s) used ChatGPT in order to review language. After using this tool/service, the author(s) reviewed and edited the content as needed and take(s) full responsibility for the content of the publication.

## CRediT authorship contribution statement

**R. Toledo-Perona:** Writing – review & editing, Writing – original draft, Methodology, Investigation, Formal analysis, Data curation. **Á. Gómez-Martín:** Writing – review & editing, Writing – original draft, Visualization, Validation, Supervision, Resources, Project administration, Methodology, Investigation, Funding acquisition, Conceptualization. **A. Contreras:** Writing – review & editing, Visualization, Validation, Supervision, Software, Investigation, Formal analysis, Data curation. **M. Toquet:** Writing – review & editing, Visualization, Validation, Supervision, Methodology, Investigation, Data curation. **J.J. Quereda:** Writing – review & editing, Visualization, Supervision, Methodology, Investigation, Funding acquisition. **A. Esnal:** Writing – review & editing, Validation, Supervision, Resources, Methodology. **P. González-Torres:** Writing – review & editing, Validation, Supervision, Software, Resources, Formal analysis, Data curation. **J. Gomis:** Writing – review & editing, Writing – original draft, Visualization, Validation, Supervision, Methodology, Investigation, Conceptualization.

## Declaration of competing interest

The authors declare the following financial interests/personal relationships which may be considered as potential competing interests: Á. Gómez-Martín reports financial support was provided by Spain Ministry of Science and Innovation. J.J. Quereda Torres reports financial support was provided by Spain Ministry of Science and Innovation. R. Toledo-Perona reports financial support was provided by Generalitat of Valencia. Á. Gómez-Martín reports financial support was provided by CEVA Santé Animale. R. Toledo-Perona reports financial support was provided by CEVA Sante Animale. Á. Gómez-Martín reports a relationship with CEVA Sante Animale that includes: speaking and lecture fees. R. Toledo-Perona reports a relationship with CEVA Sante Animale that includes: speaking and lecture fees. If there are other authors, they declare that they have no known competing financial interests or personal relationships that could have appeared to influence the work reported in this paper.

## Figures and Tables

**Figure:**
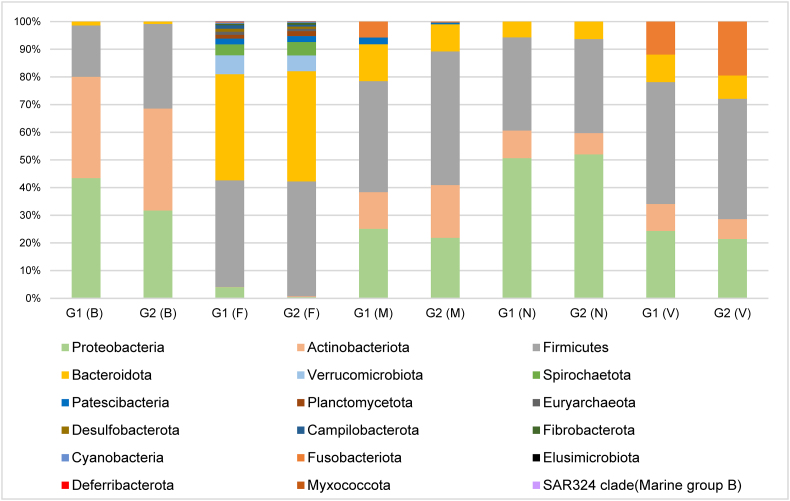
Supplementary Figure S1. Relative abundance of taxa at the phylum level depending on the experimental group (G1 or G2). Only the taxa with a mean relative abundance >1% for the different samples are shown. B, blood; F, faeces; M, milk; N, nasal; V, vaginal.

## Data Availability

Data or models are deposited an available in an official repository (PRJEB82843).

## Appendix A Supplementary data

Supplementary material 1 Image 1
