# Supplementary material for: Metabarcoding analysis of the microbiota in flocks naturally infected by Coxiella burnetii: First description of the global microbiota in domestic small ruminants
Source: One Health. 2025 Feb 19;20:100996. doi: 10.1016/j.onehlt.2025.100996 (PMC11908555; doi:10.1016/j.onehlt.2025.100996)
Supplement: Supplementary file 1 — Supplementary material 1 [file mmc1.docx]

**Metabarcoding analysis of the microbiota in flocks naturally infected by *Coxiella burnetii*: First description of the global microbiota in domestic small ruminants.**

R. Toledo-Perona^a^, Á. Gómez-Martín^a,*^, A. Contreras^b^, M. Toquet^a^, J.J. Quereda^c^, A. Esnal^d^, P. González-Torres^a,e^, J. Gomis^a^

*animal* journal

**Supplementary Table S1.** Number of phylotypes detected depending on the herd and the type of sample.

|  | **Herd A** | **Herd B** | **Herd C** | **Goat vs. Sheep** |
| --- | --- | --- | --- | --- |
| Milk samples | 707 | 614 | 2534 | 3670 |
| Faecal samples | 2061 | 3152 | 3064 | 7566 |
| Blood samples | -^1^ | 122 | 644 | 784 |
| Nasal samples | 350 | 598 | 1120 | 1929 |
| Vaginal samples | 913 | 508 | 1574 | 2863 |
| Males samples^2^ | 1204 | 1237 | 1073 | - |
| Environmental samples^3^ | 500^3^ | 712 | 951 | - |

^1^It was not possible to obtain due to the statistical analysis. ^2^Preputial, nasal, faeces and blood samples. ^3^Herd A: bedding and troughs samples; herd B and C: bedding, troughs and domestic animal’s samples.

**Supplementary Table S2.** Median (interquartile interval) of the alpha diversity indices of faecal, milk, blood and vaginal microbiota of females from each flock.

| **Comparation** | **Alpha diversity metric** | | **Faecal** | **Milk** | **Nasal** | **Blood** | **Vaginal** |
| --- | --- | --- | --- | --- | --- | --- | --- |
| Herd A | Richness (Observed OTU´s) | *P*-value | 0.24994 | 0.0662 | 0.77648 | - | 0.22757 |
|  |  | G1 | 247.00 | 28.00 | 54.00 | - | 45.00 |
|  |  | G2 | 347.50 | 91.50 | 39.00 | - | 152.00 |
|  | Evenness (Pielou index) | P-value | 0.3534 | 0.94817 | 0.17771 | - | 0.63768 |
|  |  | G1 | 0.89 | 0.90 | 0.64 | - | 0.69 |
|  |  | G2 | 0.90 | 0.93 | 0.50 | - | 0.69 |
| Herd B | Richness (Observed OTU´s) | *P*-value | 0.01984* | 0.66693 | 0.67057 | 0.32968 | 0.42072 |
|  |  | G1 | 378.00 | 78.00 | 56.00 | 13.00 | 36.50 |
|  |  | G2 | 260.00 | 57.50 | 65.50 | 14.50 | 77.00 |
|  | Evenness (Pielou index) | *P*-value | 0.62636 | 0.00013* | 0.09548 | 0.81231 | 0.10473 |
|  |  | G1 | 0.90 | 0.90 | 0.83 | 0.77 | 0.66 |
|  |  | G2 | 0.91 | 0.94 | 0.81 | 0.48 | 0.43 |
| Herd C | Richness (Observed OTU´s) | *P*-value | 0.20697 | 0.53187 | 0.57789 | 0.00176* | 0 |
|  |  | G1 | 449.50 | 308.00 | 127.50 | 25.00 | 32.50 |
|  |  | G2 | 419.50 | 351.00 | 151.50 | 78.00 | 256.00 |
|  | Evenness (Pielou index) | *P*-value | 0.02863* | 0.51383 | 0.54378 | 0.0869 | 0.16786 |
|  |  | G1 | 0.90 | 0.78 | 0.64 | 0.25 | 0.68 |
|  |  | G2 | 0.91 | 0.94 | 0.71 | 0.38 | 0.75 |
| Female groups | Richness (Observed OTU´s) | *P*-value | 0.56761 | 0.86276 | 0.93978 | 0.1674 | 0.00003* |
|  |  | G1 | 379.00 | 134.50 | 54.50 | 10.50 | 34.00 |
|  |  | G2 | 348.00 | 92.00 | 68.50 | 8.50 | 173.00 |
|  | Evenness (Pielou index) | *P*-value | 0.79463 | 0.37989 | 0.49614 | 0.63152 | 0.77316 |
|  |  | G1 | 0.92 | 0.90 | 0.80 | 0.64 | 0.68 |
|  |  | G2 | 0.90 | 0.93 | 0.73 | 0.71 | 0.61 |
| Animal specie | Richness (Observed OTU´s) | *P*-value | 0.0269* | 0.07949 | 0.00005* | 0.00514* | 0.95452 |
|  |  | Goat | 313.50 | 85.00 | 38.50 | 7.00 | 78.50 |
|  |  | Sheep | 406.00 | 153.00 | 73.00 | 12.00 | 52.00 |
|  | Evenness (Pielou index) | *P*-value | 0.01801* | 0.45936 | 0.00496* | 0.57144 | 0.68142 |
|  |  | Goat | 0.90 | 0.90 | 0.67 | 0.68 | 0.69 |
|  |  | Sheep | 0.92 | 0.92 | 0.80 | 0.63 | 0.65 |

**P* < 0.05.

**Supplementary Table S3.** FDR-adjusted *P* values of pairwise comparisons using PERMANOVA analysis for beta diversity indexes matrices in the three comparisons (between herd; between experimental groups G1 *vs.* G2; between species).

| **Type of sample** | **Group comparison** | **Qualitative indexes** | | **Quantitative indexes** | |
| --- | --- | --- | --- | --- | --- |
|  |  | Jaccard | Unweighted UniFrac | Bray Curtis | Weighted UniFrac |
| Faecal | A_ G1 *versus* A_G2 | 0.636 | 0.704 | 0.602 | 0.66 |
|  | B_ G1 *versus* B_G2 | 0.222 | 0.033* | 0.331 | 0.019* |
|  | C_ G1 *versus* C_G2 | 0.379 | 0.074 | 0.115 | 0.034* |
|  | G1 *versus* G2 | 0.321 | 0.416 | 0.44 | 0.611 |
|  | Goat *versus* Sheep | 0.001* | 0.001* | 0.001* | 0.001* |
| Milk | A_ G1 *versus* A_G2 | - | - | - | - |
|  | B_ G1 *versus* B_G2 | - | - | - | - |
|  | C_ G1 *versus* C_G2 | 0.128 | 0.119 | 0.103 | 0.031* |
|  | G1 *versus* G2 | 0.304 | 0.618 | 0.198 | 0.148 |
|  | Goat *versus* Sheep | 0.21 | 0.116 | 0.21 | 0.072 |
| Nasal | A_ G1 *versus* A_G2 | - | - | - | - |
|  | B_ G1 *versus* B_G2 | 0.053 | 0.015* | 0.022* | 0.234 |
|  | C_ G1 *versus* C_G2 | 0.08 | 0.034* | 0.081 | 0.305 |
|  | G1 *versus* G2 | 0.31 | 0.112 | 0.046* | 0.546 |
|  | Goat *versus* Sheep | 0.001* | 0.001* | 0.001* | 0.005* |
| Blood | A_ G1 *versus* A_G2 | - | - | - | - |
|  | B_ G1 *versus* B_G2 | - | - | - | - |
|  | C_ G1 *versus* C_G2 | 0.571 | 0.08 | 0.046* | 0.031* |
|  | G1 *versus* G2 | 0.936 | 0.703 | 0.963 | 0.888 |
|  | Goat *versus* Sheep | 0.026* | 0.001* | 0.017* | 0.022* |
| Vaginal | A_ G1 *versus* A_G2 | 0.031* | 0.173 | 0.492 | 0.758 |
|  | B_ G1 *versus* B_G2 | 0.285 | 0.247 | 0.682 | 0.779 |
|  | C_ G1 *versus* C_G2 | 0.028* | 0.032* | 0.131 | 0.316 |
|  | G1 *versus* G2 | 0.005* | 0.001* | 0.356 | 0.466 |
|  | Goat *versus* Sheep | 0.029* | 0.023* | 0.046* | 0.073 |

A, herd A; B, herd B; C, herd C; G1, group 1(aborted females); G2, group 2 (females with normal delivery). Values with * *P* < 0.05.

|  | **Blood** |  | **Faecal** |  | **Vaginal** |  | **Nasal** |  | **Milk** |  |
| --- | --- | --- | --- | --- | --- | --- | --- | --- | --- | --- |
|  | Group | *P-value* | Group | *P-value* | Group | *P-value* | Group | *P-value* | Group | *P-value* |
| **Phylum** |  |  |  |  |  |  |  |  |  |  |
| Proteobacteria | C, G1>G2 | <0.05 | A, G1>G2 | <0.01 |  |  |  |  | A, G1>G2 | <0.05 |
| Firmicutes | C, G1<G2 | <0.05 |  |  | C, G1<G2 | <0.01 |  |  | C, G1>G2 | <0.05 |
| Campilobacterota |  |  | B, G1>G2 | <0.01 |  |  |  |  |  |  |
| Spirochaetota |  |  | C, G1>G2 | <0.05 | C, G1<G2 | <0.05 | C, G1<G2 | <0.05 |  |  |
| Actinobacteria |  |  |  |  |  |  | C, G1>G2 | <0.05 | A, G1<G2 | <0.05 |
| **Genus** |  |  |  |  |  |  |  |  |  |  |
| *Treponema* |  |  | C, G1>G2 | <0.05 |  |  |  |  |  |  |
| *Acinetobacter* |  |  |  |  |  |  | A, G1<G2 | <0.01 |  |  |
| *UCG-003* |  |  | C, G1<G2 | <0.05 |  |  |  |  |  |  |
| *UCG-001* |  |  | B, G1>G2/ C, G1<G2 | <0.05  <0.01 |  |  |  |  |  |  |
| *UCG-004* |  |  | C, G1>G2 | <0.05 |  |  |  |  |  |  |
| *Staphylococcus* |  |  |  |  | A, G1<G2 | <0.05 | B, G1<G2 | <0.01 | C, G1>G2 | <0.01 |
| *Jeotgalicoccus* |  |  |  |  | B, G1>G2 | <0.05 |  |  |  |  |
| *UCG-010* |  |  | C, G1>G2 | <0.01 | C, G1<G2 | <0.05 |  |  |  |  |
| *RC9 gut group* |  |  | C, G1<G2 | <0.05 |  |  |  |  |  |  |
| *Prevotella* |  |  | B, C, G1<G2 | <0.05 |  |  |  |  |  |  |
| *Alistipes* |  |  | B, G1<G2 | <0.05 |  |  |  |  |  |  |
| *Muribaculaceae* |  |  | B, G1>G2 | <0.01 |  |  |  |  |  |  |
| *Campylobacter* |  |  | B, G1<G2 | <0.01 |  |  |  |  |  |  |
| *NK4A214 group* |  |  |  |  |  |  |  |  | A, G1>G2 | <0.01 |
| *Pelomonas* |  |  |  |  |  |  |  |  | A, G1>G2 | <0.05 |
| *Bacteroides* |  |  |  |  |  |  |  |  | C, G1<G2 | <0.01 |
| *Corynebacterium* |  |  |  |  |  |  |  |  | C, G1<G2 | <0.05 |
| *Salinicoccus* |  |  |  |  |  |  | B, G1<G2 | <0.05 | C, G1<G2 | <0.01 |
| *Streptococcus* |  |  |  |  |  |  |  |  | C, G1<G2 | <0.05 |
| *Mannheimia* |  |  |  |  |  |  | B, G1>G2 | <0.05 |  |  |
| *Weissella* |  |  |  |  |  |  | B, G1<G2 | <0.01 |  |  |
| **Species** |  |  |  |  |  |  |  |  |  |  |
| *Staphylococcus equorum* |  |  |  |  |  |  | C, G1<G2 | <0.05 |  |  |
| *Moraxella ovis* |  |  |  |  |  |  | B, G1>G2 | <0.01 |  |  |
| *Leuconostoc citreum* |  |  |  |  |  |  | B, G1>G | <0.05 |  |  |

**Supplementary Table S4.** Description of the taxa (phylum/genus/species level) with significant differences between experimental groups (G1 and G2) from each herd or between general groups comparation.

A: herd A; B: herd B; C: herd C; G1: aborted females; G2: non-aborted females.

**Supplementary Table S5**. Description of the taxa (phylum/genus/species level) with significant differences between species (goat or sheep) and general groups comparation (G1-G2).

|  | **Blood** |  | **Faecal** |  | **Vaginal** |  | **Nasal** |  | **Milk** |  |
| --- | --- | --- | --- | --- | --- | --- | --- | --- | --- | --- |
|  | Group | *P-value* | Group | *P-value* | Group | *P-value* | Group | *P-value* | Group | *P-value* |
| **Phylum** |  |  |  |  |  |  |  |  |  |  |
| Proteobacteria |  |  | G1>G2 | <0.01 | G<S | <0.01 |  |  |  |  |
| Actinobacteriota | G>S | <0.05 | G1<G2 | <0.01 |  |  |  |  |  |  |
| Bacteroidota |  |  | G<S | <0.05 |  |  |  |  |  |  |
| Proteobacteria |  |  | G>S | <0.05 |  |  |  |  |  |  |
| Firmicutes |  |  | G>S | <0.01 |  |  |  |  |  |  |
| Campilobacterota |  |  | G1<G2 | <0.01 |  |  |  |  |  |  |
| Patescibacteria |  |  | G>S | <0.05 |  |  |  |  |  |  |
| Spirochaetota |  |  |  |  |  |  |  |  |  |  |
| **Genus** |  |  |  |  |  |  |  |  |  |  |
| *Corynebacterium* | G<S | <0.05 |  |  |  |  | G<S | <0.01 | G>S | <0.05 |
| *Candidatus Saccharimonas* |  |  | G>S | <0.05 |  |  |  |  |  |  |
| *Bacteroidales RF16 group* |  |  | G<S | <0.05 |  |  |  |  |  |  |
| *Prevotellaceae UCG-003* |  |  | G<S | <0.05 |  |  |  |  |  |  |
| *Staphylococcus* |  |  |  |  |  |  |  |  | G<S | <0.01 |
| *Escherichia-Shigella* |  |  |  |  | G<S | <0.01 |  |  |  |  |
| *Campylobacter* |  |  | G1<G2 | <0.05 |  |  |  |  |  |  |
| *Salinicoccus* |  |  |  |  |  |  |  |  | G1<G2 | <0.01 |

G, goat; S, sheep; G1: aborted females; G2: non-aborted females.
